# Review of Advances in Coating and Functionalization of Gold Nanoparticles: From Theory to Biomedical Application

**DOI:** 10.3390/pharmaceutics16020255

**Published:** 2024-02-09

**Authors:** Wilmmer Alexander Arcos Rosero, Angelica Bueno Barbezan, Carla Daruich de Souza, Maria Elisa Chuery Martins Rostelato

**Affiliations:** Instituto de Pesquisas Energéticas e Nucleares, São Paulo 05508-000, Brazil; wilmmer.rosero@ipen.br (W.A.A.R.); angelbbarbezan@gmail.com (A.B.B.); carlepy@gmail.com (C.D.d.S.)

**Keywords:** nanotechnology, gold nanoparticles (AuNPs), biomolecules in nanoparticles, nanoparticle functionalization, nanotheranostics, therapeutic efficacy, biomedical applications, cell membrane coated, biocompatibility

## Abstract

Nanoparticles, especially gold nanoparticles (Au NPs) have gained increasing interest in biomedical applications. Used for disease prevention, diagnosis and therapies, its significant advantages in therapeutic efficacy and safety have been the main target of interest. Its application in immune system prevention, stability in physiological environments and cell membranes, low toxicity and optimal bioperformances are critical to the success of engineered nanomaterials. Its unique optical properties are great attractors. Recently, several physical and chemical methods for coating these NPs have been widely used. Biomolecules such as DNA, RNA, peptides, antibodies, proteins, carbohydrates and biopolymers, among others, have been widely used in coatings of Au NPs for various biomedical applications, thus increasing their biocompatibility while maintaining their biological functions. This review mainly presents a general and representative view of the different types of coatings and Au NP functionalization using various biomolecules, strategies and functionalization mechanisms.

## 1. Introduction

Nanotechnology, resulting from technological advances in microscopy, involves the manipulation of matter at a nanometric scale, very close to quantum limits. Although the analysis of archaeological objects has revealed the presence of nanoparticles in decorative items, the boom in nanotechnology research occurred at the end of the past century, addressing questions about obtaining nanomaterials. In databases, there are thousands of articles on nanotechnology, covering synthesis, characterization and applications in various fields from medicine to geology. On a nanometric scale, matter exhibits characteristics distinct from those on a macroscopic scale, forming the basis for significant technological advances [1,2].

Nanoparticles are typically presented as colloids, consisting of a solid phase and a liquid phase, usually water or an organic solvent, due to the methods of obtaining them. Two main routes for obtaining nanoparticles are top–down and bottom–up. The former is primarily of a physical nature, such as laser ablation, while the latter is mainly a chemical route, starting with molecules that interact to produce stable nanometric systems with specific characteristics [3].

Colloids are of great interest in various research areas due to their intrinsic properties, including a high surface-to-volume ratio, facilitating interactions and increasing reactivity and functionalization possibilities of nanomaterials. 

**Figure 1:**
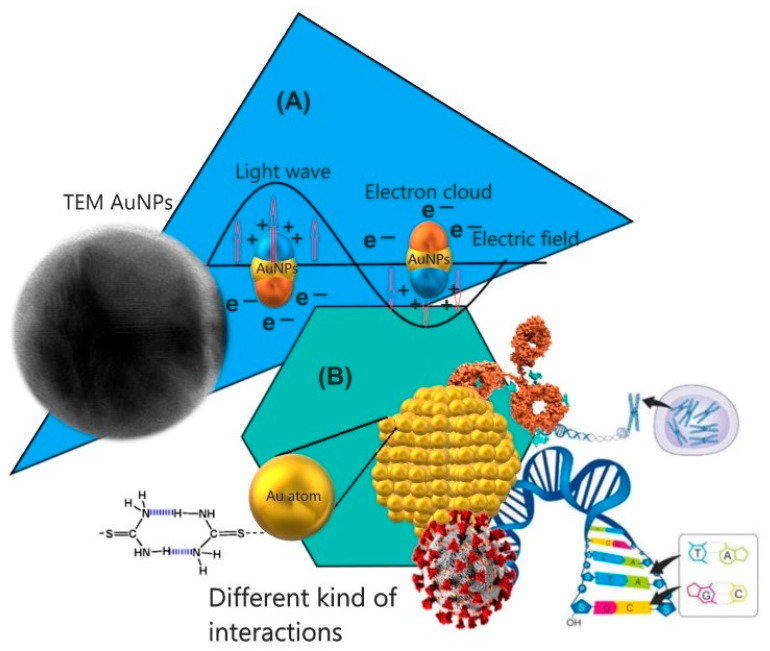
(**A**) Surface plasmon resonance (SPR) phenomenon involving what happens on the surface of gold nanoparticles when interacting with light. (**B**) Some types of coatings used for gold nanoparticles, ligands, medicinal agents, DNA, antibody, etc. These interact through covalent-type ligations such as S-Au or electrostatic, and at the same time molecules linked to gold atoms can form interactions such as H-H hydrogen bridges.

The optical properties resulting from surface plasmon resonance in metallic nanomaterials have found applications in a wide spectrum of technological and medical applications. When illuminated by light, metal particles undergo a coherent collective oscillation of free electrons in response to the oscillating electromagnetic field of the light. This electronic oscillation around the particle surface induces a charge separation with the ionic lattice, resulting in a dipole oscillation aligned with the electric field of light (Figure 1A), which can favor electrostatic interactions of the dipole–dipole type. This phenomenon, known as surface plasmon resonance (SPR), manifests as a maximal amplitude oscillation at a specific frequency. Notably, SPR in plasmon nanoparticles, particularly those composed of noble metals like Au and Ag, exhibits a significantly stronger absorption of incident light compared to other metals. Measurement of SPR can be conducted using a UV-Vis absorption spectrometer. The intensity and wavelength of the SPR band are influenced by factors affecting electron charge density on the particle surface, including metal type, particle size, shape, structure, composition and the dielectric constant of the surrounding medium, as elucidated by Mie theory [4].

Metallic nanoparticles in colloidal form, particularly iron oxide, gold and silver, have attracted attention due to their versatility, ease of synthesis and stability [5]. Au NPs, in particular, are extensively studied for medical applications, as they can be coupled with various functionalizing moieties, including ligands, medicinal agents, DNA, amino acids, proteins, peptides and oligonucleotides, through different types of interactions (Figure 1B). Numerous studies have demonstrated that Au NPs enter organelles and blood vessels to reach the tumor site, reflecting the extensive research in this area [1].

Innovative nanotechnologies are transforming clinical practices, particularly in diagnosing, treating and managing human diseases [6]. These technologies focus on the nanoscale drug delivery system, which ensures targeted and efficient drug delivery. Active targeting is achieved by integrating a ligand specific to the disease’s receptor or epitope [7].

Key to these nanotechnologies is their biocompatibility and biodegradability. This ensures that, once the drug is delivered, the nanocarrier breaks down into harmless components, facilitating its safe removal from the body [6].

Upon administration, nanoparticles undergo biodistribution, crossing epithelial barriers and circulating through the vascular system. Smaller particles (<20–30 nm) are typically excreted renally, while larger ones may be absorbed by the mononuclear phagocytic system (MPS) in organs like the liver and spleen [8].

The clearance rates of these particles are influenced by factors such as endothelial fenestral pore sizes, age, sex and genetics. This variability presents challenges in assessing nanomedicine efficacy and toxicity across different individuals [6].

Finally, the uptake of nanocarriers by macrophages is regulated through opsonization, a process mediated by the innate immune system [9].

Currently, nanoparticle-based therapeutic systems, exemplified by Doxil, have gained traction in the pharmaceutical market. Doxil, a widely used chemotherapeutic drug, employs a polyethylene glycol (PEG) liposome delivery system. While liposomes dominate the landscape of FDA-approved nanotechnology drugs, recent attention has shifted toward inorganic materials characterized by a high atomic number (Z). In the realm of radiotherapy, these materials aim to enhance the local radiation dosage while minimizing damage to healthy tissues [2,10].

Research on lymphatic administration of liposomal formulations dates to the 1940s, with J.Y. Johnson pioneering the development of artificial phospholipid vesicles, known as liposomes, for pharmaceutical industry applications [11].

In the following years, various researchers proposed similar methods for liposome creation, particularly emphasizing medical applications. This period marked significant advances, especially when compared to gold nanoparticles, an area that has recently seen increased research activity.

Between 2019 and 2021, there was a notable reduction in publications on gold nanoparticles in biomedical applications, an impact attributed to the COVID-19 pandemic. However, in 2022, with the easing of restrictions and the search for alternatives to combat the virus, a resurgence in research involving AuNPs was observed.

Many of the recent studies utilizing AuNPs as a vehicle are discussed in this review. Despite the increased focus on gold nanoparticles, their biomedical applications continue to show promising prospects.

Comparing methods for obtaining liposomes with gold nanoparticles (AuNPs), we find that liposomes spontaneously form through the amphiphilic properties of phospholipids, creating bilayers [12]. Although obtaining AuNPs is not inherently complex, it requires specific conditions. A significant advantage of AuNPs is the multitude of published synthesis methods, many with ecological considerations [13,14].

Liposomes and AuNPs offer numerous advantages, each showcasing diverse applications and commercialized products, reflecting variations in research timelines. Notably, a critical limitation of AuNPs is their cytotoxicity, which depends on particle size, morphology, environmental conditions and production methods [15]. In contrast, liposomes present versatile targeting options, employing both active and passive techniques to focus on specific tissues.

The clearance of liposomes by the mononuclear phagocytic system (MPS), due to their size range of 400–5000 nm, is a notable challenge. PEGylation helps mitigate opsonization, thereby reducing MPS clearance and extending their circulation half-life. 

Gold nanoparticles (Au NPs) are recognized as the most stable metal nanoparticles, exhibiting unique properties not found in macroscopic materials. Their nanometric form allows for various advantageous features, especially in detection applications where intrinsic nanoparticle properties are utilized for intelligent sensors [16,17]. The synthesis of AuNPs is relatively straightforward, with versatility in altering size and shape through synthesis parameter modifications [16,17].

An additional noteworthy characteristic of AuNPs is their large surface-to-volume ratio. This facilitates the immobilization of substantial quantities of specific functional groups, resulting in swift responses and enhanced sensitivity to the target analyte. The plasmon resonance in AuNPs can be easily modified, allowing for variable optical properties tailored to specific needs. Moreover, nanoparticles exhibit excellent chemical and biological compatibility with active molecules, making them ideal for transport scaffolding with minimal impact on the functional activity of their active coating. This versatility extends to applications in colorimetric, fluorimetric, spectroscopic and electrochemical methods, positioning AuNPs as multifaceted detection platforms [2,16,18,19].

The diverse applications of AuNPs are dictated by their unique characteristics. The ability to couple with a wide array of functionalizing moieties, including ligands, therapeutic agents, DNA, amino acids, proteins, peptides and oligonucleotides, has fueled interest in their medical applications. Extensive research indicates that AuNPs not only traverse blood vessels to reach tumor locations but also penetrate organelles. The inefficient removal of AuNPs from tumors, attributed to poor lymphatic drainage in malignant tissues, enhances their passive accumulation. This underscores the potential of AuNPs as ‘magic bullets’, broadening their scope of applications [1,10,19,20,21].

## 2. Materials and Methods

This survey aims to bring together a research bank in a single location, with a prevailing reference to the scientific literature that emerged in the past 10 years. For this purpose, a systematic literature search was carried out in the databases of the following platforms: ScienceDirect, Google Scholar and Web of Science. 

The keywords used were the following:

- Coating of Gold Nanoparticles;
- Encapsulating of Gold Nanoparticles;
- Encapsulation of Gold Nanoparticles;
- Capped of Gold Nanoparticles;
- Cell membrane camouflaged of Gold Nanoparticles.

Our exhaustive investigation spanned from 2014 to 2023, yielding over 255,000 studies related to Au NPs. In an endeavor to enhance research quality, we conducted a subsequent search, employing article relevance as a parameter. This refined search yielded an additional 400 scientific studies.

**Scheme 1:**
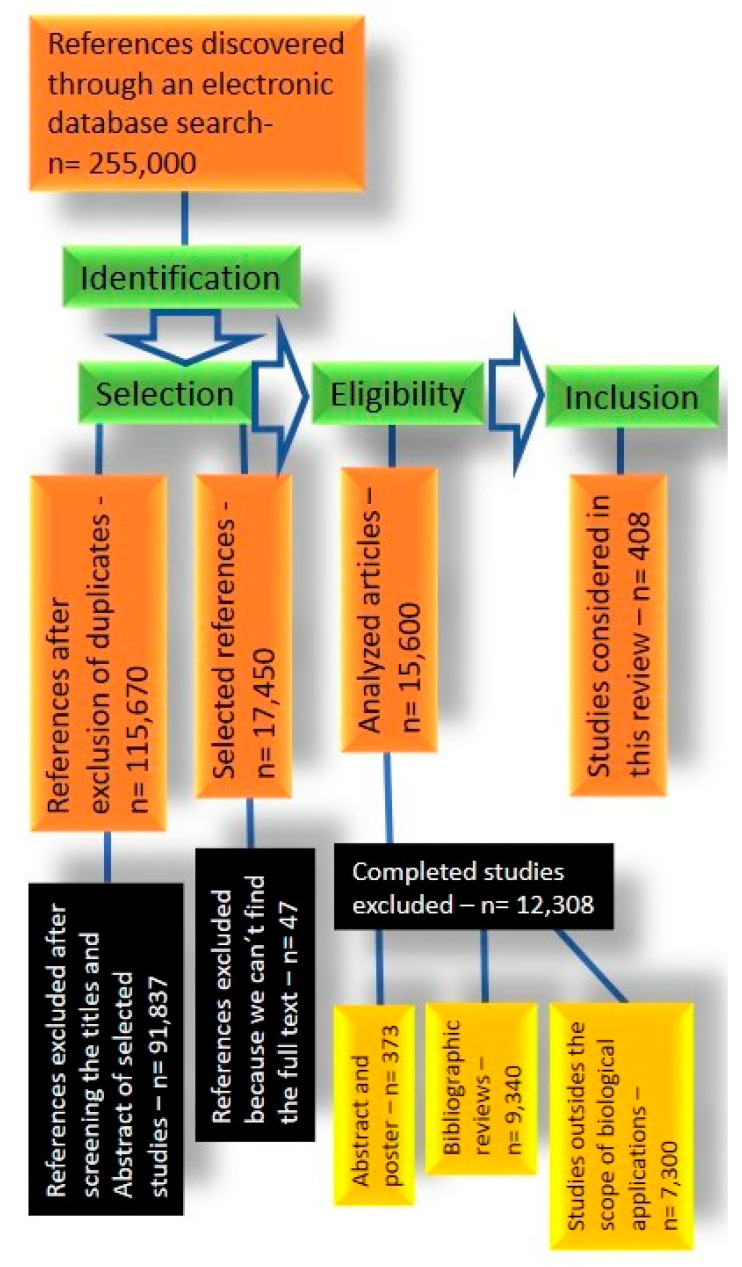
Flowchart for Inclusion of Studies Performed.

This extensive undertaking stems from the imperative to elucidate the interplay between nanoparticles (the fundamental unit in nanotechnology) and cells (the basic unit in biology). This comprehension is crucial for advancing diagnostic and therapeutic techniques across various pathologies. A profound understanding of the interface between nanotechnology and biology is essential for achieving superior outcomes in medical applications (Scheme 1).

## 3. Literature Review on Gold Nanoparticles

### 3.1. Synthesis of Gold Nanoparticles

**Figure 2:**
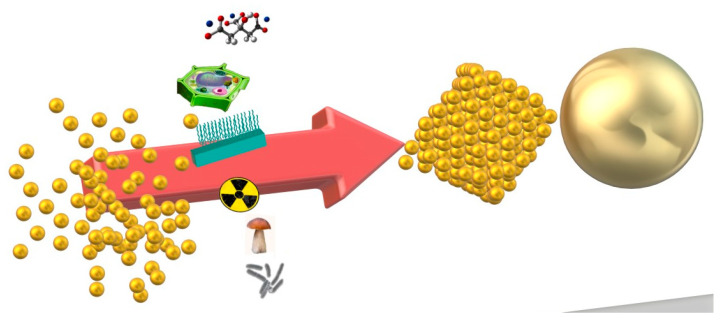
Exploring diverse routes in bottom–up nanoparticle synthesis: from chemical techniques to eco-friendly biological and radiative methods. In the bottom–up approach, there is a wide variety of routes, completely chemical methods such as the Turkevich method and variants, biological methods where microorganisms such as fungi are used, often called eco-friendly, and methods of obtaining the use of gamma radiation.

Two primary approaches exist for synthesizing gold nanoparticles (AuNPs), “Top-Down” and “Bottom-Up” [1,10], as illustrated in Figure 2. Similar to many other kinds of inorganic nanomaterials, top–down or bottom–up methods could be used to create AuNP. When using a top–down strategy, physical techniques like laser ablation, aerosol technology, UV and IR irradiation and ion sputtering are used to convert a bulk gold into AuNP. Contrarily, the bottom–up method of synthesizing AuNP begins at the atomic level (gold ions) and works its way up to nanoparticles of the required size and shape by using the right chemistries. The reduction of Au ions with the appropriate reducing agents in the presence of capping agents is the key step in the chemical process used to prepare spherical AuNP. 

The “Top-Down” approach entails the synthesis of AuNPs from bulk materials, breaking them down into nanoparticles through various methods.

The “Bottom-Up” method, the focus of this review, initiates nanoparticle synthesis from the atomic level. It comprises two primary stages:

1. Reduction of a gold precursor, typically a salt in an aqueous solution. The choice of reducing agent, ranging from citrate to sodium borohydride, directly influences nanoparticle size [1,10];
2. Stabilization of the AuNPs, achieved through coatings forming covalent bonds or electrostatic stabilization. The selection of coating depends on the intended application and its interaction with the target [1,10,17,22].

In contrast, the “Top-Down” approach, although intuitive, may yield AuNPs with variations in size and shape, potentially being less cost-efficient. Conversely, the “Bottom-Up” approach provides precise control over AuNP properties and is more economical for large-scale production. However, its optimization can pose challenges due to synthesis variables. This review focuses on the “Bottom-Up” approach due to its relevance in biomedical applications, where nanoparticle specificity and functionalization are crucial [1,10].

### 3.2. Methods of Synthesis of Au NPs

Over the years, various methods have been employed, each with its unique features:

- Turkevich Method;
- Brust Method;
- Seed-Mediated Growth;
- Digestive Ripening;
- Martin Method;
- Radiation;
- Biological Synthesis.

Currently, the pathways for synthesizing gold nanoparticles (AuNPs), as illustrated in Figure 2, are broadly understood, encompassing a detailed grasp of the variables influencing the outcome of the AuNPs and their corresponding mechanistic perspectives. Widely acknowledged chemical protocols for AuNP synthesis include the Frens/Turkevich method (yielding 10–100 nm hydrophilic spherical AuNPs), the Brust method (producing 1–3 nm hydrophobic spherical AuNPs), the Murphy/El-Sayed surfactant-assisted seed-mediated method (employed for gold nanorods) and the polyolgalvanic method (used in preparing gold hollow polyhedral nanoparticles). Additionally, modified methods rooted in green chemistry principles are also documented in the literature.

Collectively, AuNPs derive advantage from the existence of well-optimized, repeatable and tunable synthetic methodologies, enabling the generation of a diverse library of AuNP with varying sizes and shapes through accessible chemical processes. Notably, the current landscape facilitates the acquisition of AuNP from diverse industrial providers, offering a spectrum of options in terms of size, shape and surface chemistry [1,2,3,5,6].

- Turkevich Method (1951)

This widely used method creates spherical AuNPs by reducing gold ions (Au^3+^) to gold atoms (Au^0^) using various reducing agents, like citrate or UV radiation. A variant involves sodium borohydride (NaBH_4_) for a simplified approach without heating [1,10,22].

- Brust Method (1994)

Utilizing a two-phase reaction with organic solvents, this method yields AuNPs ranging from 1.5 to 5.2 nm. It employs sodium borohydride and alkanethiol, with Tetraoctylammonium bromide facilitating the phase transfer [1,10,22].

- Seed-Mediated Growth

Predominantly used for creating nanometric gold rods, this method leverages pre-synthesized particles, which subsequently grow with weak reducing agents [1,10].

- Digestive Ripening

Optimal for producing monodisperse AuNPs, this method relies on temperature modulation, heating colloidal suspensions up to 138 °C and then cooling them [1,10].

- Martin Method

Emphasizing stoichiometry control, this approach adjusts the ratio of reactants for optimal nanoparticle synthesis. This method allows AuNPs to combine with hydrophilic molecules for various applications [23,24].

- Radiation

Employing radiation influences the synthesis of AuNPs. The radiation dose rate determines the size of the nanoparticles, with higher rates leading to smaller sizes [5].

- Biological Synthesis

An eco-friendly approach uses living organisms, from bacteria to plants and fungi, for AuNP synthesis. This method stands out for its environmental benefits and cost-effectiveness [1,10,22].

- Other Methods

Various other techniques, like the use of bacteria, fungi, plants, algae and biomolecules, also contribute to the broad landscape of gold nanoparticle synthesis. Each method, whether using bacterial cell walls, fungal secretions, plant components or algae biomass, offers unique pathways for producing AuNPs with distinct properties [1,10,22].

## 4. Coating and Functioning

Due to the often overlapping and sometimes ambiguous terminology associated with nanoparticle studies, it is necessary to clarify two central terms: ‘coating’ and ‘functionalization’. This section aims to dispel any doubts regarding these terms, ensuring a clear and distinct understanding of each within the context of this work. Gold nanoparticles are extensively researched for their unique properties and potential applications in therapy, diagnostics and technology. Modifying these intrinsic properties can be achieved through two main processes, coating and functionalization, each with its nuances and objectives.

Coating involves encapsulating or covering the nanoparticle’s surface with a specific material. The primary goal is to provide stability, protect against degradation and optimize dispersion in specific media. Materials such as polymers (e.g., PEG), surfactants and organic entities serve as protective barriers, promoting a more harmonious integration of nanoparticles with their introduced environment.

In contrast, functionalization refers to the intentional modification of the nanoparticle’s surface to confer a desired functionality. This process is typically achieved through the covalent bonding of specific molecules or functional groups to the nanoparticle’s surface. Such modifications enable nanoparticles to respond to specific stimuli, bind to defined targets (such as cells or proteins) or serve as carriers for therapeutic agents. An illustrative example is the attachment of antibodies to nanoparticles, directing them selectively to tumor cells.

In practice, many applications initially require a coating to ensure the stability and biocompatibility of nanoparticles. Subsequently, additional functionalization is carried out to instill specific functionalities. Together, these techniques enable the customization of gold nanoparticles, optimizing their therapeutic efficacy and diagnostic capability. This study will delve into both processes in detail, shedding light on their intricacies and implications [25].

### 4.1. Applications and Functionalization of Coated AuNPs

The use of coated gold nanoparticles (AuNPs) has rapidly expanded in recent years, primarily due to their ability to bind to a variety of molecules, offering diverse applications in biomedicine. Furthermore, functionalization plays a pivotal role in nanoparticle design. By attaching a specific target molecule to the surface of the AuNPs, it is possible to direct the nanoparticle to a specific organ or tumor tissue. This approach has the potential to enhance treatment efficacy by reducing side effects, as the drug is delivered directly to the target site.

#### 4.1.1. Drug Delivery

Chemotherapeutic agents, as drugs, inhibit cell replication and induce apoptosis, thereby limiting cellular function. This elimination capacity extends to healthy cells, restricting the use of certain drugs due to their impact on healthy tissues. Consequently, extensive research focuses on developing selective drugs to minimize collateral damage [22,26,27].

The expanding body of research has enhanced our comprehension of various physicochemical properties, including size, surface charge and their modifications, influencing the cellular absorption and fate of Au NPs. Notably, larger nanoparticles tend to accumulate near the vasculature, while smaller ones rapidly diffuse throughout the tumor matrix. The optimal size range for Au NPs in tumor treatment falls between 25 and 50 nm, depending on the tumor type. Regarding charge, it plays a crucial role; Au NPs with a positive charge exhibit 5 to 10 times greater absorption than their negative or neutral counterparts. This is attributed to their high affinity with negatively charged cell surfaces, facilitating enhanced adhesion to the cell membrane through the generation of transient holes [20,28].

**Figure 3:**
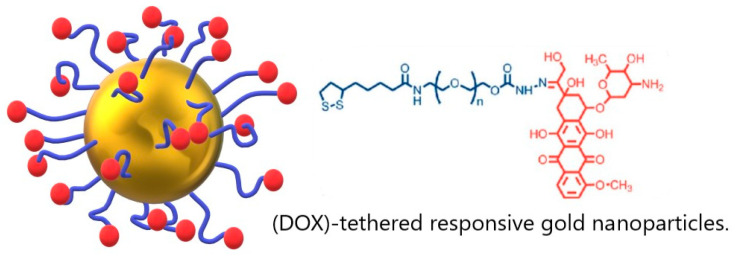
Schematic illustration of doxorubicin (DOX)-tethered responsive gold nanoparticles.

As drug carriers, as depicted in Figure 3, gold nanoparticles (Au NPs) transport therapeutic agents through either covalent bonding or non-covalent attachment. Covalent bonding requires the chemical modification of drugs and external triggers for drug release, while non-covalent adsorption onto Au NPs provides an alternative method for efficient drug transport and release [28].

Due to their potential applications as delivery vehicles, diagnostic tools and therapeutic agents, gold nanoparticles are among the most extensively studied metallic nanomaterials. The extensive surface area of GNPs makes them well suited for functionalization with biomolecules via physical absorption or with reactive groups via ionic or covalent bonding for ligand or antibody modification. In general, GNPs are biocompatible and have low toxicity, and they have good biocompatibility [29].

Considering the goals of cancer therapy, which include achieving greater absorption, drug permeability, site specificity and control over the drug release rate, the role of Au NPs as drug chaperones is pivotal. Numerous studies suggest that, depending on the objective, Au NPs can incorporate specific functional groups to aid in orientation, exhibit fluorescence or possess imaging capabilities to trace their path [2,10,30].

The Retention and Permeability Effect (RPE) refers to the improper formation of vasculature, resulting in reduced pore size. This facilitates passive directional transport to tumor vasculature, enabling Au NPs to bypass natural body barriers. Consequently, Au NPs evade degradation or drug metabolism, allowing accumulation in tumor regions. The limited lymphatic drainage in these areas further prevents nanoparticle recirculation.

Numerous advantages of nanoparticles in transporting chemotherapeutic drugs have been elucidated, including a significant increase in drug circulation, inhibiting rapid elimination by the liver and kidneys [2,10,30].

Another strategy for Au NPs in drug delivery involves leveraging active targeting mechanisms. This entails binding specific ligands, such as peptides, aptamers or antibodies, to the Au NPs’ surface. The inclusion of these ligands ensures preferential binding to target cell receptors, serving as recognition moieties. This highly specific targeting minimizes effects on healthy tissue, thereby enhancing treatment efficacy [27,30,31].

#### 4.1.2. Au NPs in Immunotherapy

Oncological immunotherapy relies on harnessing the patient’s immune system to combat neoplasms [32], recognized as a highly effective approach in cancer treatment. Interest is growing in delivering immunotherapeutic agents through nanoparticles, with Au NPs emerging as potent vectors for this purpose [32]. Due to their advanced surface chemistry, Au NPs can carry various antigens and adjuvants, enhancing the immune response against tumor cells and inducing cytotoxic T lymphocytes. These nanoparticles can mimic antigen-presenting cells by being functionalized with co-stimulatory molecules and proteins containing antigenic peptides, enabling interaction with dendritic cells and enhancing cytokine production, positioning them as promising adjuvants [32,33,34].

In oncological vaccinology, the current trend focuses on activated dendritic cells [35], where Au NPs serve as effective tools for delivering therapeutic vaccines [36,37]. Studies indicate that Au NPs coated with tumor antigens promote dendritic cell maturation and subsequent lymphocyte proliferation [38]. The induced modifications in dendritic cells, including phagocytosis, activation, migration and T-cell co-stimulation, are fundamental for the success of vaccines based on these cells [32,33,34,35,36,37,39].

Experimental data highlight the relevance of Au NPs in immunological interactions, showcasing their antigenic, adjuvant and pro-inflammatory properties. Thus, the future of combined oncological therapy, integrating phototherapy, chemotherapy and immunotherapy, may be shaped by the strategic development of multifunctional nanoparticles, with a particular emphasis on those based on Au NPs [32].

#### 4.1.3. Au NPs as Radiosensitizers

Gold nanoparticles (AuNPs), with their elevated atomic number, present an enlarged X-ray absorption cross-section, rendering them optimal candidates as radiosensitizers to enhance radiotherapy efficacy. Additionally, the photothermal conversion capability of AuNPs can generate localized heat within the tumor region [2,10,20].

The versatile biomedical applications of AuNPs encompass the detection of cancer-associated biomarkers. Yang et al. devised a sandwich-structured electrochemiluminescence (ECL) sensor for detecting phosphatidylserine-positive exosomes, crucial in an early ovarian cancer (OC) diagnosis. This ECL sensor featured glassy carbon electrodes coated with gold “nanoflowers” (AuNFls) and a signal nanoprobe comprising graphitic carbon nitride sheets loaded with luminol-coated gold nanoparticles (Lum-AuNPs@g-C3N4). The high specific surface area of AuNFls and g-C3N4 facilitated the initial modification of abundant specific binding peptides (FNFRLKAGAKIRFRGC) of phosphatidylserine onto these materials [40].

Another application involves a rapid colorimetric method using gold nano-probes designed for detecting OC-associated miRNAs. In this method, miRNAs formed pairs with complementary AuNPs-miRNA probes, resulting in AuNP aggregation and a shift in the absorption spectrum.

Moreover, fluorescence resonance energy transfer (FRET) probes, utilizing carbon quantum dots as energy donors and AuNPs as energy acceptors, were developed for highly sensitive CA125 detection, a clinical marker for OC [40].

The localized intracellular presence of AuNPs heightens the likelihood of ionization events, increasing the accumulation of local energy and causing more harm to tumor cells. The distinction in mass energy absorption coefficients between gold and soft tissues, enabling dose escalation, underlies the physical mechanism of AuNP radiosensitization within the initial nanoseconds of exposure. Electrons emitted from AuNPs exhibit a limited range of a few micrometers, resulting in very localized ionizing events. Therefore, precise delivery to and absorption by tumor cells during radiotherapy are imperative to realize any benefits [2].

The chemical mechanism of AuNP radiosensitization involves radiochemical sensitization of DNA, mediating an increase in catalytic surface activity and augmenting free radical generation on AuNPs’ surface. Contrary to previous belief, the electronically active surface of AuNPs has been demonstrated to catalyze chemical reactions, primarily through interaction with molecular oxygen, producing free radicals. These catalytic effects, in combination with radiation, appear to enhance [2].

Radiobiological mechanisms contributing to cellular response to AuNP irradiation include the generation of reactive oxygen species (ROS), oxidative stress, induction of DNA damage, potential bystander effects and impacts on the cell cycle. Oxidative stress can result in the oxidation of lipids, proteins and DNA, leading to necrotic, apoptotic or other forms of cell death. Elevated ROS levels also affect mitochondrial activity, depleting cellular energy and causing necrotic death [2].

## 5. Stabilization of AuNPs

### 5.1. Electrostatic Stabilization

van der Waals forces, leading to system instability and precipitation, induce the aggregation of nanoparticles in colloidal systems. Numerous techniques have been employed since the inception of nanomaterial research to stabilize nanoparticles and ensure electrostatic stability. As per the Derjaguin–Landau–Verwey–Overbeek hypothesis, the total potential energy governing the interaction between two colloidal particles comprises both attractive (van der Waals) and repulsive forces arising from the electrical double layer of charges [1,41,42,43].

A particle is deemed stable if its total potential energy surpasses the kinetic energy. However, defining nanoparticle stability solely in terms of electrostatic stabilization is insufficient; surface energy must also be considered. Metallic surfaces, in conjunction with attractive dipole–dipole interactions, typically exhibit surface energies ranging from 1000 to 2000 mJm^−2^, markedly higher than those observed in other organic and inorganic materials, contributing to the instability of these nanomaterials [5,10].

**Figure 4:**
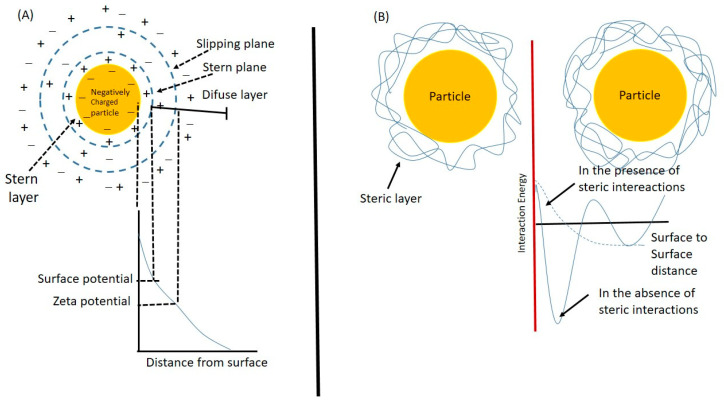
Electrostatic and steric repulsion model. (**A**) Electrostatic stabilization of AuNPs is based on Coulomb repulsion between particles with the same surface charge sign, preventing the particles from coming into close contact where the attractive short-distance van der Waals forces dominate. (**B**) For steric stabilization, the contact is avoided by adsorbing bulky molecules on the surface, which act as a spacer to prevent particle aggregation [1,44].

A charged layer can form on the colloidal nanoparticle surface when ionic groups from the liquid dispersion medium adhere to it. Consequently, the colloidal nanoparticles are enveloped by an equal number of ions with opposing charges, forming electroneutral double layers. Figure 4 illustrates this stabilization through an electrical double layer generated by conflicting pressures, inhibiting nanoparticle aggregation in the solution phase through electrostatic repulsions. Electrostatic stabilization can be controlled by adjusting key factors, including pH, concentration and temperature [1].

An alternative method of stabilization involves combining steric and electrostatic approaches to maintain the stability of metallic nanoparticles in the solution phase. Polyelectrolytes, when used as polymeric surfactants, integrate steric and electrostatic stability effects into a single molecule. An ionic surfactant with extended end chains and a polar head group creates a two-fold electric layer around the nanoparticle, providing steric repulsion within the particles. This configuration prevents agglomeration, resulting in a mutual stabilization system. Figure 4 elucidates how steric and electrostatic interactions combine to stabilize AuNPs [1].

### 5.2. Steric Stabilization

**Figure 5:**
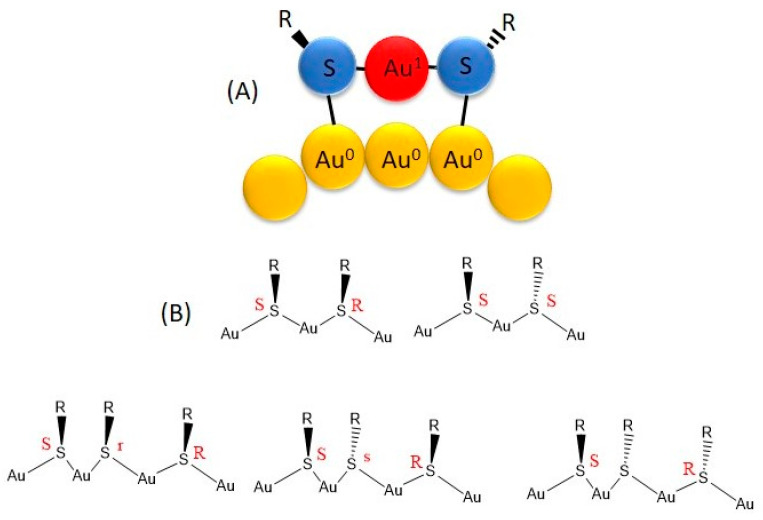
Stereochemistry in protecting units on the surface of gold clusters. (**A**) Schematic drawing of a monomeric Au–SR–Au staple motif. (**B**) Stereochemistry in protecting units on the surface of gold clusters. While the gold atoms stabilized between two sulfur atoms are formal Au, the gold atoms at the bottom of the structures are Au^0^ atoms at the surface of the cluster core (I). The stereodescriptors are indicated in red, and lowercase letters indicate pseudochirality. Priority for the determination of absolute configuration is Au^0^ > Au^1^ > R > electron lone pair (schematic and image taken from “Chirality in Thiolate-Protected Gold Clusters”) [46].

Steric stabilization (Figure 5B) is employed to uphold the stability of nanoparticles through the attraction or repulsion of adsorbed ions or molecules amidst adjacent particles. Generally, enhanced steric stabilization is achieved with larger adsorbed molecules. A variety of functional groups, such as hydroxyls, surfactants, oligomers and polymers, are utilized to accomplish this type of stabilization. The spatial conformation of molecules plays a crucial role, and it has been demonstrated that elongated or conical conformations contribute to greater stability. Notably, if the size of the nanoparticle is smaller than that of the stabilizer, typically observed with long polymer chains, an encapsulation process can occur, resulting in the passivation of the particle [1,45].

## 6. Functionalization of Au NPs

When selecting Au NPs for biological applications, careful consideration must be given to the interaction medium. The complexity of a biological medium, as evidenced by in vitro tests, is heightened when introduced into a living organism.

Within the body, prior to reaching the target, Au NPs undergo compositional changes. When injected into the bloodstream, they interact with serum proteins, forming a coating known as the corona protein. At this stage, the corona dictates the biological functions of Au NPs, including internalization, biodistribution, elimination and potential toxicity. Various pathways, such as endocytosis mediated by specific or nonspecific receptors or the enhanced effect of permeability and retention, enable Au NPs to enter cells [10,18].

Functionalizing Au NPs for drug delivery or diverse applications often involves non-covalent interactions for drug loading. Alterations in native physical forces are sufficient for release, without necessitating specific bond cleavage. Hydrophobic drug release can be achieved by inducing changes in local hydrophobicity. Covalent ligation with the drug, forming cleavable bonds through internal or external incentives, presents another alternative.

To enhance binding to biological molecules and optimize Au NPs as drug carriers with increased specificity, certain functionalization techniques employ synthetic pathways containing functional residues. Utilizing one or more functional groups, such as oligo or polyethylene glycol (PEG), bovine serum albumin (BSA), amino acids, polypeptides, oligonucleotides, antibodies, receptors and similar particles, is common. A widely adopted method for attaching fractions, such as chemotherapeutic drugs, involves employing biomolecules with thiol groups [2,45].

Research on self-assembled monolayers (SAMs) has deepened the understanding of a widely used coating agent for gold nanoparticles. Chemically, sulfur is softer and less electronegative than oxygen, exhibiting a greater number of oxidation states and a stronger affinity for metals on the right in the d block. Gold, in particular, displays a significant affinity for sulfur [45,46].

Studies on the crystal structure of thiolate-coated gold clusters have uncovered stable SR(-Au-SR) motifs, seemingly crucial for the stability of Au-S bonds in thiol-stabilized Au NPs. In these systems, gold atoms are intercalated between sulfur atoms of thiolates, presenting several stable motifs, as illustrated in Figure 5, where it demonstrates that sulfur atoms in stable structures act as stereogenic centers, attached to four different substituents: two gold atoms, the organic R part of the thiolate, and a lone pair of electrons. Initially, the organic part R of the thiolate can assume two positions, with easy interconversion due to a low barrier [45,46].

With an increasing number of studies, additional stable motifs in gold clusters protected by thiolates have been discovered. Structural determinations reveal trimeric SR(-Au-SR) motifs, thiolate bridges, cyclic structures or even bare sulfur atoms. Structural studies indicate that the Au-S interface is dynamic, involving processes like thiolate migration, thiolate desorption and re-adsorption, contributing to flexibility [45,46].

Two primary techniques exist for functionalizing Au NPs with specific molecules. An efficient approach entails synthesizing Au NPs directly with the molecule of interest linked to a thiol group, as previously discussed. This method offers the mentioned advantages. Alternatively, a second approach involves post functionalization of Au NPs. Click chemistry and the succinimidyl ester reaction have both been employed to covalently bind molecules of interest to the ligand stabilizing Au NPs. Alternatively, ligand exchange may be utilized if the molecule of interest possesses a thiol group [47].

### 6.1. Protein Corona Formation

Nonspecific targeting and immune system activation are notable challenges in Au NP drug delivery. Coating affected cells with PEG, as proposed in some studies, resolves these issues by concealing the cell surface, inhibiting surface protein attachment, and diminishing the likelihood of immune system activation. However, a flawlessly coated surface hinders nanoparticle adhesion to specific receptors, rendering them “invisible” to the immune system. To address this, gold nanoconjugates undergo further modification with specific ligands to navigate in vivo challenges, despite potential side effects. Incorporating functional moieties on the particle surface facilitates the attraction of specific “stealth” proteins, forming a protein corona that shields the nanoparticles from phagocytic cells [48].

Understanding the intricacies of the immune system is crucial to overcoming challenges like those mentioned above. While certain Au NPs demonstrate promising in vitro results, the biological complexity of in vivo systems necessitates a comprehensive understanding and anticipation of the formation of the so-called crown protein for the successful application of Au NPs in biological contexts.

**Figure 6:**
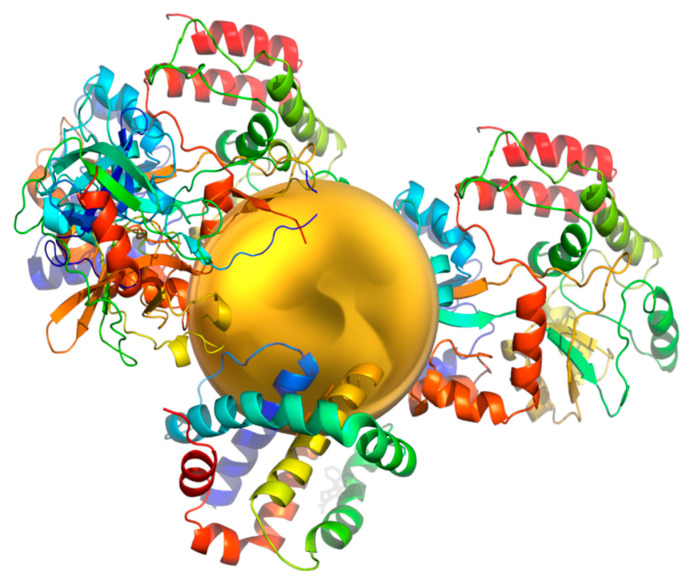
Au NP protein corona complex. The affinity and exchange rate of the protein crown depend on the size, charge, composition, geometry, incubation conditions (temperature, concentration, time), the type of proteins and their stability. The formation of the crown defines the cellular internalization, biodistribution and toxicity of the particle [47,49,50,51].

In the body, proteins adsorb onto the surface of Au NPs, leading to the formation of a dynamic protein crown, a process vividly illustrated in Figure 6. This protein coating is subject to ongoing competitive interactions until an equilibrium state is reached. Initially, a rapid formation of proteins creates the ‘soft crown’. Over time, proteins with a higher affinity for the surface gradually replace the initial layer, culminating in the formation of a more stable ‘hard crown’. As a result, both the composition and the thickness of this protein crown are subject to dynamic changes over time.

The composition of the protein–nanoparticle complex undergoes continuous changes during the nanoparticle’s interaction with the body. Initially, it is highly probable that proteins characterized by elevated plasma concentrations and robust association rates will occupy the nanoparticle’s surface. Over time, these proteins may disassociate, making room for others with lower concentrations, slower exchange rates or stronger affinities. This phenomenon, encompassing the competitive adherence of proteins to a finite surface based on abundance, affinities and incubation time, is commonly referred to as the “Vroman Effect” [52,53]. This effect holds considerable significance concerning the distribution of particles within the body. The evolution of the protein corona, occurring as the nanoparticle traverses different body compartments during its circulation, may be influenced by fluctuations in protein levels and binding affinities [54,55].

This holds particular relevance for nanoparticles due to the heightened importance of surface effects at this scale. Numerous studies have demonstrated that biological responses to nanoparticles exhibit a stronger correlation with surface area than with mass. Nanoscale materials possess significantly higher surface-to-volume ratios compared to larger particles, as surface areas decrease more gradually than volumes with diminishing size. Consequently, a nanoparticle with a higher surface-to-volume ratio is expected to bind a greater quantity of proteins in comparison to its mass, as supported by existing research [54,55].

The impact of varying protein binding on the biological response to a specific nanoparticle remains uncertain, as does the process of protein binding (e.g., uptake by phagocytic cells of the reticuloendothelial system and clearance). However, it is evident that the type and quantity of proteins present on a particle’s surface influence the biodistribution of nanoparticles. A comprehensive understanding of the kinetics, affinities and stoichiometries of protein attachment and dissociation from the nanoparticle, as well as the specific proteins associated with the particle, is crucial for a thorough comprehension of the protein corona [56].

The predominant focus of research investigating the impact of protein binding on absorption has involved preincubating particles with bulk serum/plasma, preincubating particles with specific proteins or attaching individual proteins to the particle’s surface, followed by the analysis of uptake by macrophages. This research consistently reveals a direct correlation between surface charge and protein binding, with neutrally charged particles opsonizing at a substantially slower pace than charged particles [9,57].

### 6.2. PEGylation of Au NPs

Poly (ethylene glycol) (PEG) contributes to a widely accepted and clinically approved coating strategy, renowned for its efficacy in prolonging blood circulation half-life to optimize pharmacokinetics. This stealth capability arises from the rapid chain motion and large excluded volume of PEG, inducing steric repulsion upon binding to foreign substances. PEG’s low interfacial free energy in water facilitates the easy synthesis and stabilization of PEG-coated nanoparticles (NPs) in aqueous media. Previous studies have underscored the significance of PEG coating density and chain length as key determinants influencing the stealth quality of PEG coatings [58].

Despite being generally considered non-toxic, PEG functions as an anchored hydrophilic polymer when used to functionalize Au NPs. Thiol-terminated PEG, commonly possessing biotin, nitriloacetic acid, -COOH, OH or -OCH3 end groups, allows the conjugation of various biomolecules, such as proteins [59,60].

While PEG imparts greater stability to Au NPs in vivo and mitigates undesirable immune responses, its usage diminishes cellular uptake compared to citrate stabilized Au NPs. This drawback can be overcome by co-functionalizing the Au NP surface with moieties like receptor-mediated endocytosis (RME) peptides, enhancing cellular uptake while preserving stability and immune response characteristics achieved through PEG modification [61,62].

**Figure 7:**
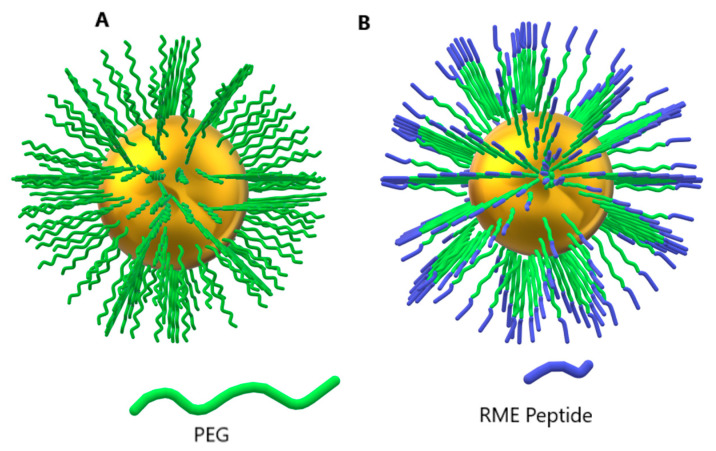
Co-functionalization of AuNPs typically employs two techniques. The first method entails producing a mixed monolayer, for instance, by directly adhering PEG and RME to the surface of AuNP using thiol chemistries (**A**). The second method makes use of a hetero-bifunctional PEG linker, such as thiol-PEGsuccinimidyl glutaramide (SH-PEG-SGA), which has an NHS ester group at one end for attachment to the AuNP surface and a thiol at the other end for direct binding to the RME peptide via the N-terminus group (**B**). Therefore, the latter configuration has the ability to fully saturate the AuNP surface with PEG before saturating the free end of PEG with a physiologically active group.

The widespread application of PEG in the functionalization of Au NPs, primarily due to its easy adaptability with various molecules, is clearly demonstrated in Figure 7. Recognized for its clinical approval for intravenous use, the amphiphilic nature of PEG contributes to the stabilization of nanoparticles in biological environments. It is important to note that Au NPs are often not exclusively bound to PEG. As shown in Figure 7B, they can be linked to PEG either before or after binding to other fragments such as peptides, oligonucleotides or different molecules tailored to specific applications [1,61].

Consequently, PEG-Au NPs find utility not only in cellular functionalization but also in intracellular internalization. Studies indicate that PEG inhibits agglomeration and cytotoxicity of Au NPs in high-ion-concentration environments, supporting prolonged circulation and increased utility of Au NPs. The hydrophilic corona formed by PEG on the Au NP surface significantly reduces nonspecific protein adsorption, evading opsonization and complement activation in the blood. Notably, PEG coatings may, however, negatively impact drug delivery efficacy, potentially reducing cellular uptake and endosomal release of therapeutics loaded in the NPs, and diminishing NP tumor targeting efficacy [1,63].

To achieve complete surface saturation with peptides or other biologically active groups, a hetero-bifunctional PEG linker is proposed, potentially enhancing direct cell/peptide contact with the target cell. However, concerns have been raised regarding the stability compromise and anti-PEG effects associated with PEG linkers [64].

The degree of PEGylation alters basic physiochemical properties of Au NPs, influencing uptake by immune cells. Particle size and PEG density-dependent adsorption of serum proteins by PEGylated Au NPs impact the efficiency of uptake by macrophages [60].

Data on critical coagulation concentrations (CCCs) indicate that PEG length, nanoparticle diameter and the PEG molecule/nanoparticle ratio influence nanoparticle stability in high-ionic-strength media. Longer PEGs, smaller nanoparticles and higher PEG/nanoparticle ratios favor more stable sols, aligning with DLVO theory and Flory–Krigbaum theory predictions [65].

Considering the crucial role of diameter in biomolecule–nanoparticle conjugates, especially for cell nucleus access, PEGs of different sizes (900, 1500 and 5000) increase the hydrodynamic radius of 20 nm gold nanoparticles by 3.5, 5 and 9 nm, respectively. PEG 5000, while enhancing stability, may compromise nuclear translocation effectiveness due to its additional bulk [65].

Studies have demonstrated that PEGylated nanoparticles resist removal by the reticuloendothelial system, with extracted nanoparticles from the systemic circulation accumulating in the liver after payload release [66].

PEGylation encompasses a broad range of sizes and classes within the PEG molecule. Typically, another molecule is covalently linked, with its purpose depending on the desired application. As presented in Table 1, diverse molecular systems supported on PEG have been reported for functionalizing Au NPs.

### 6.3. Antibody and Protein Functionalized Au NPs

The active targeting of nanoparticles to known oncogenes prompted the immediate consideration of antibodies as the optimal choice for this purpose. Antibodies exhibit a robust affinity for specific receptors, rendering them highly selective. Examples of gold nanoparticles (Au NPs) frequently employed for enhanced radiotherapy in targeting malignancies include cetuximab and trastuzumab. Receptor-mediated endocytosis facilitates the uptake of antibodies into cells; however, the conjugation of antibodies is known to alter their pharmacokinetics, leading to distinct subcellular localizations between the antibody and the Au NPs. This process provides a target-specific internalization mechanism for antibodies-Au NPs. Post-conjugation, alterations in the antibody’s kinetics and absorption are anticipated. While documented examples suggest favorable changes for improved absorption, further research is warranted in this area [17,91].

**Figure 8:**
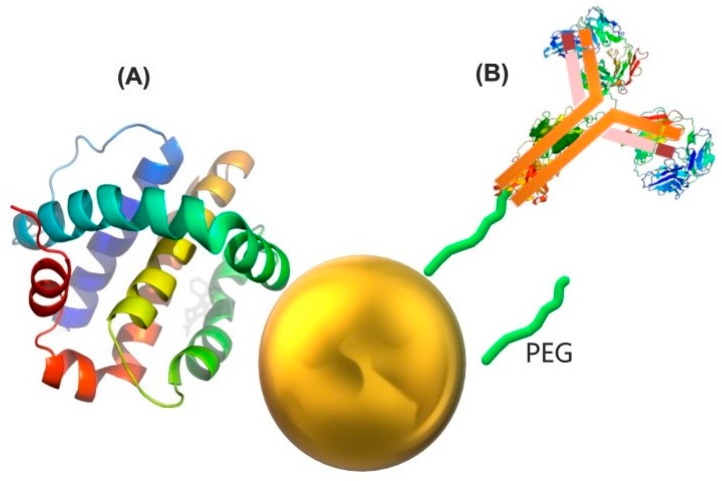
Antibody and protein functionalized Au NPs. (**A**) Coating of gold nanoparticles with proteins by means of electrostatic interactions. Protein binding association constants on AuNPs increase progressively with AuNP diameter between 5 and 60 nm. The binding association constant, on the other hand, was discovered to be slowly varying for some AuNPs with diameters greater than about 80 nm [99]. (**B**) Functionalization of antibodies by means of a PEG bridge. Every year, more antibodies are used to functionalize Au NPs. Most of these systems are stabilized with thiolated linkers like PEG chains, which are typically terminated in carboxylic acids or succinimidyl esters that are then activated for the addition of antibodies by forming random amide bonds with free amine residues on the surface of the antibodies.

To cross-link Au NPs with carboxylic acids on antibody surfaces, a hydrazine-terminated PEG linker can be employed (Figure 8B). However, the literature predominantly utilizes some variant of amide bond formation involving lysines on antibodies, given the higher abundance of carboxylic acids than amines. This chemistry tends to be relatively random, generating more variants [17].

Random amide bond formation may result in bonds forming at or near the antibody’s active site or orienting the active site toward the gold nucleus, diminishing conjugate attachment. To optimize activity, the orientation of antibodies on an Au NP surface can be adjusted using protein G [17].

While antibodies exhibit indisputable affinity for receptors, their size may impede tumor penetration. An alternative approach involves using antibody fragments. Functional antibody fragments are produced by reducing disulfide links between an antibody’s heavy chains, exposing free thiols that can bind to the gold nucleus. This method positions the antibody’s active site away from the nucleus, enhancing accessibility [17,92].

The physical interaction between antibodies and gold nanoparticles is determined based on three phenomena: (a) ionic attraction between positively charged antibodies and negatively charged gold; (b) hydrophobic attraction between antibodies and the gold surface; and (c) dative binding between gold-conducting electrons and amino acid sulfur atoms of antibodies. Various methods facilitate chemical interactions, including (i) chemisorption using thiol derivatives, (ii) the use of bifunctional linkers and (iii) the use of adapter molecules such as streptavidin and biotin [93].

While spontaneous protein-Au NP adsorption is recognized, limited information is available on the structural and molecular details of this interaction (Figure 8A). Recent studies indicate reduced activity of enzymes like lysozyme and chymotrypsin upon adsorption to Au NPs, with a similar effect observed for fibrinogen. Pepsin remains active when attached to Au NPs, and adsorption enhances the stability of bovine catalase at high temperatures. CD tests reveal that although enzyme activity may diminish on the nanoparticle surface, proteins seem to retain their secondary structure. Therefore, the effect of enzyme activity retention after adsorption appears to be protein-specific [94,95].

In comparison to other targeted therapies, the utilization of native proteins as targeted agents remains largely unexplored. Natural receptor ligands or lectins, proteins binding to carbohydrates often extracted from fruits and vegetables, serve as targeting moieties. However, the abundance of competing ligands in the human body lacking Au NPs may reduce selection effectiveness. This underscores the need for further exploration in this area [17,96,97,98].

The modification of Au NPs or the promotion of self-assembly can be achieved using thiols and protein scaffolds. An alternative approach involves the direct adsorption of proteins onto the Au NP surface, exemplified by the “crown protein” (Au NP CP), which garners considerable attention. This entity, governed by biological dynamics, binding affinity and protein adsorption rate, crucially influences the exchange time and lifetime of interfacial interactions. Investigations reveal that the corona protein presence enhances surface free energy, and proteins can be conjugated with Au NPs through glutamic acid. This involves bonding with amino groups and carboxyl groups extending outward, forming connections with protein amino groups [1,98].

Interaction between Au NPs and proteins (Figure 8A) can induce intrinsic characteristic changes in both. Notably, physiological modifications such as alterations in bound protein structure, complement activation, blood clotting and protein aggregation may arise [100,101,102,103]. The hydrophilicity or hydrophobicity of Au NPs plays a regulatory role in the amount and composition of protein adsorption. For instance, hydrophilic polyethylene glycol (PEG) modification results in strong resistance to plasma proteins, such as complement subunit adsorption, enabling extended circulation time due to resistance to the reticuloendothelial system (RES) [104].

Colloidal gold nanoparticles capped with citrate attract immunoglobulins and other proteins through non-covalent interactions. These interactions involve hydrophobic attractions to the metal surface, electrostatic interactions between positively charged amino acids, the protein’s N-terminus and the negatively charged surface of citrate-capped particles [93,105]. Gold–sulfhydryl group bonds, particularly with cysteine side chains of proteins, contribute to tight, practically irreversible protein binding on the AuNP surface, especially at a pH just above their pI values [95].

Tighter protein binding may impact function and structure. Immunoglobulins, despite their large size (150 kDa), maintain antigen-binding activity when combined with AuNPs. However, smaller proteins may face inactivation risks, although some enzymes may retain functionality. Conjugation may occasionally enhance enzyme stability and activity, while protein immobilization on NP surfaces can increase tolerance to thermal and pH gradients and reduce susceptibility to denaturation and degradation [95].

To enhance Au NP dispersibility, surface treatment with electrolytes like citrate, CTAB, PSS, PDDAC, etc., is common. This alteration imparts net charges on Au NPs, leading to electrostatic attraction to functional groups in proteins with opposing charges. Fine-tuning the conjugation pH to approximately 0.5 units above the protein’s isoelectric point is essential to prevent protein aggregation due to electrostatic attraction. This adjustment maintains hydrophobic interactions, facilitating protein–gold conjugation. System optimization for specific antibodies to each Au NP preparation involves determining the optimal pH value for conjugation [105].

While recombinant proteins have been studied, many investigations employ native receptor proteins, benefiting from the advantageous properties of recombinant proteins derived from mutations and variants of wild-type proteins. Table 2 and Table 3 present antibodies and proteins used as coatings on Au NPs. Copper-pro- moted alkyne-azide cycloaddition reaction CuAAC can efficiently react the azo group on gold nanoparticles with the alkyne group on the protein surface, and a protein labeled with an alkyne-containing photoaffinity probe in solution can also be click-captured, enriched on a clickable reagent, and easily released by thiol exchange reaction [97].

### 6.4. Peptides and Amino Acid Functionalized Au NPs

**Figure 9:**
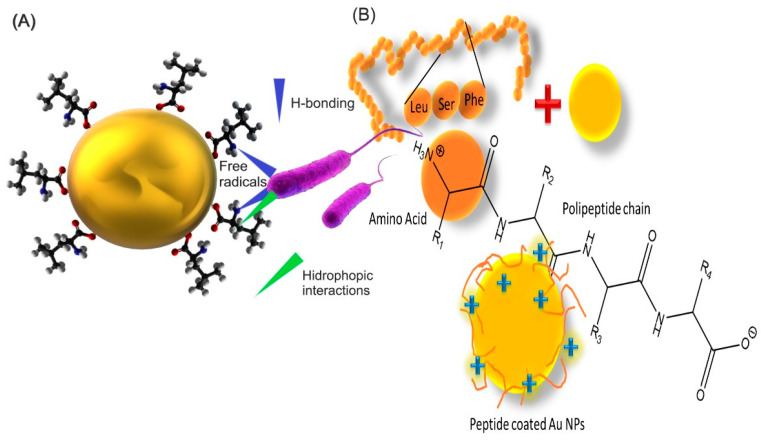
Peptides and amino acid functionalized Au NPs. (**A**) Different types of amino acid interactions with bacteria. (**B**) Nanoparticles allow easy functionalization of peptides for gene delivery, with several types of interactions between the peptide fragments being possible; stability increases [141,142].

A methodology to enhance biocompatibility involves surface engineering or custom coating of metal nanoparticles with peptides or amino acids, as depicted in Figure 9 [138].

Peptides, shorter polymers of amino acids, are chemically synthesizable and fully characterizable to specifications. Despite a lower affinity toward receptors compared to proteins, peptides have garnered significant attention due to their simplicity and rapid absorption kinetics. Their excellent biocompatibility, non-toxicity and environmental friendliness make peptides with specific sequences ideal conjugates for Au NPs. Figure 9 showcases different types of amino acid interactions with bacteria (Figure 9A) and the ease of functionalizing nanoparticles with peptides for gene delivery, including several types of interactions between peptide fragments (Figure 9B). The versatility of peptide-based Au NP systems, coupled with gentler manufacturing conditions, unique self-assembly methods and specific substrate-binding properties, enables their utilization in biosensors, drug delivery and cellular uptake [1,96].

Peptides, spanning 0.4 to 1 nm and comprising 2 to 50 amino acids, facilitate cell-specific targeting when bound to target molecules on specifically designed carriers, leveraging the nanoparticles’ increased reactive surface area. Their notable attributes include high stability (long shelf life), substantial carrier capacity (incorporating multiple drug molecules in the particle matrix), the ability to carry both hydrophilic and lipophilic molecules and facilitating hassle-free drug administration through various routes [139,140].

Amine groups in amino acids and peptides, negatively charged, bind to negatively charged Au NPs, stabilizing them as negatively charged carboxylic groups expand outward. Some amino acids, such as lysine or glycine, effectively conjugate to DNA without inducing cytotoxicity. Modifying the peptide sequence allows for influencing the assembly and disassembly of Au NPs [1]. Amino acid conjugated nanoparticles show different interactions with bacteria (Figure 9A). The amino acid conjugated to the nanoparticle can interact through hydrogen bonding between the (N) of amine in amino acid and (O) of the cell surface carboxyl group. Hydrophobic interaction occurs between carbon chains of amino acid and a methyl group on the bacterial cell wall. These interactions can lead to free radical generation, which can cause membrane perturbations (pores in the membrane). Due to perturbations, there is oozing out of the bacterial cytoplasm, leading the cell to death due to loss of proteins, nucleic acids and cell structural rigidity [141,142]. 

Organic inhibitors, featuring hetero atoms, aromatic rings and conjugated systems, stand out as highly effective components in industrial applications [14,143,144]. These inhibitors serve as additives for the modification of sol–gel coatings. Notably, amino acids, characterized by their environmentally friendly nature, affordability and purity exceeding 99%, represent a compelling inhibitor class. The corrosion-inhibiting ability of amino acids stems from their propensity to establish hydrogen bonds with oxide or hydroxide groups on metal surfaces [14,143,144].

Amino acids exhibit intrinsic biological properties, such as antibacterial effects and enhanced viability for eukaryotic cells. The particle size, nanoparticle core and specific amino acid employed in surface functionalization influence biodistribution and host immunity [142]. Single amino acids provide both amino and carboxy-terminal functionalities for biomolecule conjugation. Biocompatible amino-acid-based coatings address challenges related to size, biodistribution, immune cell interaction and inflammation induction, and offer heightened biocompatibility [142].

The implementation of peptide coatings has enabled precise control over the aggregation of Au NPs. The thiol–gold interaction facilitates the anchoring of positively charged peptides, containing cysteine residues, neutralizing negative charges that might otherwise induce aggregation. Additionally, peptides disperse negatively charged Au NPs [96,97]. This innovative approach yields more stable Au NPs, simplifying the conventional citrate-mediated synthesis. The synthesis involves mixing dilute acid and an aqueous solution of multidomain peptides (MDPs) at room temperature, with 3,4-dihydroxy-L-phenylalanine (Dopa) residues ensuring excellent biological reducibility. The method offers advantages such as low toxicity, short reaction time, high engineering efficiency and precise size control [96,97].

Peptides, being smaller than proteins or antibodies, excel as targeting agents. Their relative stability on antibodies and proteins allows for linkage after binding to Au NPs or before ligand exchange [17]. Peptide coatings enhance the physiological stability of nanoparticles, enabling programming of surface chemistry and immunomodulatory activity [145]. Cell penetration peptides (CPPs) are commonly utilized for Au NP conjugation. CPPs, known for membrane translocation capability, facilitate the delivery of therapeutic molecules, proteins, liposomes, nanoparticles, antisense molecules, siRNA and nucleic acids. Categorization of CPPs includes cationic, amphipathic and hydrophobic peptides, with cationic CPPs, rich in arginine and lysine, being extensively employed for various cellular payloads [146,147].

The physical–chemical properties of CPPs allow classification into three groups: cationic, amphipathic and hydrophobic peptides. The majority of known CPPs fall under the cationic category, primarily rich in arginine and lysine. These peptides have been widely utilized for delivering diverse payloads into cells, including proteins, nucleic acids, small molecule medicines and NPs. Classification systems for CPPs are often based on physico-chemical characteristics, such as amphipathicity or hydrophobicity [148,149].

Amino acids, featuring amine and carboxylic groups, can function as both reducing and capping agents in the creation of Au NPs. All amino acids, except cysteine, have demonstrated the capability to reduce HAuCl4 to gold NPs. Studies indicate that gold NPs with amphiphilic amino acid surfaces exhibit superior biocompatibility [150]. Table 4 shows a series of peptides and amino acids reported as coating Au NPs.

### 6.5. Microorganism, DNA, RNA and Aptameros Functionalized Au NPs

DNA serves as a functionalization tool for Au NPs and is increasingly embraced in the scientific community due to its precisely defined structures and functions, coupled with its programmable assembly process based on DNA sequence, length and structure [1,17]. Since the advent of chemical DNA synthesis, DNA oligonucleotides have evolved into indispensable biopolymers in materials science. Nearly any position in DNA is modifiable during synthesis, utilizing various functional groups. The persistence length of a duplex DNA, around 50 nm or 150 base pairs, is easily achievable through synthesis. By extending the chain in increments of 0.34 nm with the addition of a base pair, DNA manipulation with sub-nanometer precision is feasible. The high programmability of DNA has led to the creation of numerous remarkable nanostructures [164]. 

**Figure 10:**
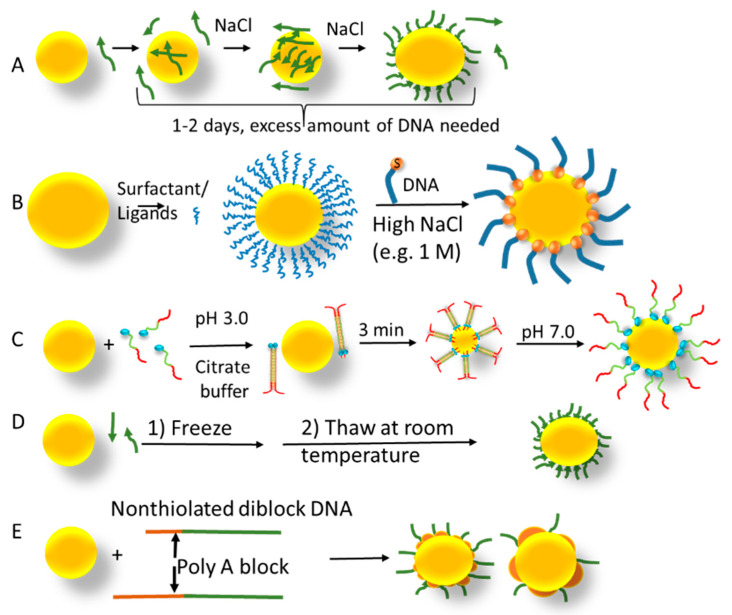
Methods for conjugating DNA on AuNPs. (**A**) The typical salt-aging process involving initial DNA adsorption, and gradual increase in NaCl concentration. (**B**) Attaching DNA in the presence of stabilizing ligands (e.g., surfactants) to improve the colloidal stability of AuNPs. (**C**) Low-pH loading requiring the DNA containing a poly(A) block to assemble into a parallel duplex. (**D**) The freezing method does not require additional reagents. (**E**) Anchoring poly(A) containing nonthiolated DNA on AuNPs with controlled density; a longer poly(A) block yields a lower DNA density (adaptation from Biwu et al.) [164].

In the realm of AuNPs, conjugation predominantly employs terminal thiol-modified DNA. This modification imparts strong electrostatic and steric stabilization from the phosphate backbone, rendering the DNA highly negatively charged (Figure 10B,C). AuNPs adorned with a dense DNA layer exhibit excellent colloidal stability. Thiolated DNA, owing to its substantial Au-S affinity (exceeding 200 kJ/mol), has long been employed for Au surface functionalization. However, a noteworthy observation is the DNA desorption noticed after prolonged storage. Intriguingly, research into the nature of the Au-S bond persists [165,166].

Controlling the separation distance between nanoparticle assemblies during synthesis allows for the creation of nanoparticle crystals, gummers or large oligomerizations. One approach involves coating the surface of Au NPs with thiol-capped single-stranded DNA molecules [1,17,96]. DNA hydrolyzing enzymes enhance the stability of DNA-Au NP complexes, with current research exploring factors influencing stabilization and techniques for achieving it [167]. Sequence specificity of DNA on Au NPs, along with the immobilization of DNA on gold improving thermal stability, has been reported [167].

Cationic-glycopolymer-stabilized Au NPs, when complexed with DNA, present a potential solution for endosomal withdrawal and nuclear uptake challenges in gene delivery. Transfection efficiencies of cationic glyconanoparticles exhibit size dependency as gene delivery aspects [168]. The stability of the double helix with Au NPs is attributed to chemical and electrostatic interactions, with low-density DNA strands potentially adopting a bent conformation for effective stabilization [169].

In the context of anticancer medications targeting DNA, understanding drug interactions with DNA is crucial for developing effective medication delivery systems. The surface density of DNA-AuNP-based sensors significantly influences their performance. At low surface densities, nonspecific binding restricts hybridization, while high densities may render capture probes ineffective, posing challenges for stability and reproducibility [170].

Storage issues, including nonspecific probe DNA contact and contamination, affect the stability of DNA-AuNPs over time. Common techniques involving gradual thiolated DNA attachment to AuNPs over 12 h require careful storage to maintain high-quality DNA-AuNPs [171]. Ionic strength, pH and local environment influence interactions between DNA and citrate-AuNPs, necessitating the addition of salt for adsorption [172].

The pH of the solution is critical for DNA-AuNP stability, with destabilization occurring below pH 5 or above pH 9. Citrate-capped AuNPs remain stable between pH 5 and pH 9, although low pH can partially protonate citrate [173,174]. Kinetic studies reveal DNA strands spreading over Au NP surfaces over time, and functionalizing gold nanoparticles with oligonucleotides for aptamer hybridization is a superior method [1,17,96].

RNA molecules offer a low-risk alternative to DNA-based treatments, enabling selective gene expression modulation. Recombinant proteins, while useful, require repeated injections and lengthy purification procedures. RNA therapies, including siRNA- and mRNA-based modulations, present a highly effective and secure alternative [175,176,177].

RNA interference (RNAi) holds potential for silencing disease-causing genes. Delivery remains a fundamental barrier, with siRNAs facing challenges in pharmacokinetics and metabolic stability. Melanomas, with constitutively activated STAT3, are targeted effectively with siRNA [178]. To extend siRNA half-life, molecular modification or carrier substances are employed [179].

Therapeutic siRNA release over time requires a biomaterial supporting storage and controlled release without negatively impacting cell viability. RNA molecules are prone to degradation and immune recognition, necessitating modification strategies for in vivo applications [180]. Aptamers, whether DNA or RNA oligonucleotides, offer advantages in cancer biomarker recognition and anticancer drug delivery [181,182].

Aptamers, being non-immunogenic and stable in various conditions, act as both targeting moieties and drug vehicles [181,183]. They demonstrate success in targeting multiple cancer cell receptors, enhancing treatment selectivity [1,96]. Aptamers address therapeutic voids similar to monoclonal antibodies, providing low immunogenicity, minimal batch variation, ease of production, prolonged shelf life, stability and targeting potential [181,184]. Aptamers also facilitate gene delivery by preventing DNase activity from reaching the target site [185]. Table 5 presents various DNA, RNA and aptamers used as coatings for Au NPs.

### 6.6. Carbohydrate Directed Au NPs

Carbohydrates play a central role in cell growth and infection events, being implicated in major infections such as HIV, tuberculosis and malaria. Glycan recognition results in the formation of lectins that selectively bind to carbohydrates. Although lectin–carbohydrate interactions exhibit weaker binding compared to specific enzyme–substrate or antibody–antigen motifs, cells overcome this limitation through multivalency. By presenting multiple glycan moieties in proximity, cells enable cooperative binding to receptors, leading to a greater increase in affinity than simple monovalent interactions. This multivalency is effectively mimicked by Au NPs, serving as excellent cell mimics with specific attributes, such as shape, size and a high specific surface area, facilitating the presentation of carbohydrates with varying spacing, ligand density and spatial orientation [198].

**Figure 11:**
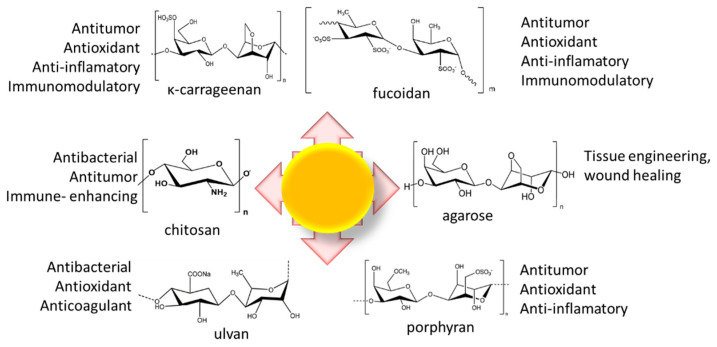
Structure and biological activity of carbohydrates that have been functionalized with Au NPs (figure is modified from Xiangyan et al.) [199].

Significant biological macromolecules, such as alginate, carrageenan, porphyran, fucoidan, ulvan, agarose and chitosan, are widely found in marine algae and animals, a variety of which are illustrated in Figure 11. These macromolecules possess desirable properties such as biocompatibility, biodegradability and lack of irritation, making them extensively useful in biomedicine and nanomaterials. Specifically, carbohydrate functionalized AuNPs, employing a greener preparation method, demonstrate enhanced biocompatibility and targeting capabilities. This is exemplified in Figure 11, where the diverse applications of these marine-derived macromolecules in AuNP functionalization are depicted [199].

In the realm of metal nanomaterials, carbohydrates, especially in AuNPs, play a crucial role. For example, the chitosan/alginate complex enhances the biocompatibility and anticancer activity of AuNPs [200]. The selective modification of carbohydrate surfaces, such as sulfation and acetylation, holds promise for improving their use in nanomaterials [199,201].

To broaden the applications of carbohydrate-based AuNP nanosystems, AuNPs can be modified using specific ligands like functional peptides and targeting receptors [202].

Non-covalent forces, including hydrogen bonds, hydrophobic interactions and van der Waals forces, facilitate the binding of carbohydrates to AuNPs. While covalent bonds are weaker, non-covalent bonds, commonly referred to as sorption or physisorption, can effectively bind carbohydrates to NPs through multiple interactions, collectively resulting in strong binding interactions [198].

They exhibit a diverse array of functional groups, such as hydroxyl, amino, carboxylate, sulfate and ester groups, amenable to functionalization through hydrogen bonding, electrostatic attraction or chemical modification [199,203].

Carbohydrates, when encapsulated, and gold nanoparticles (Au NPs), when appropriately stabilized, demonstrate prolonged blood circulation, enhancing their biomedical efficacy. These have found extensive application in drug delivery systems, mitigating adverse effects on healthy cells through electrostatic interaction, hydrogen bonding or non-covalent interaction [118,204].

Carbohydrate ligands on glyco-Au NPs play multifaceted roles in formulations, contributing to (1) increased circulation times by minimizing clearance, (2) reduced nanoparticle cytotoxicity and (3) targeting glycan-responsive receptors. Exploiting the Warburg effect, where cancer cells exhibit a preference for glucose metabolism over oxidative phosphorylation, carbohydrates can actively target cancer cells, capitalizing on the overexpression of sugar transporters in these cells. Furthermore, glycosylation of magnetic nanoparticles (MNPs) has demonstrated enhanced in vivo circulation times for NPs [198].

The distinction between cancer cells and healthy cells lies in the differential expression of lectins on their surfaces. Considering this, investigations into the affinity of carbohydrates for these lectins have been conducted to target cancer cells. Notably, molecules such as hyaluronic acid (HA), a natural polysaccharide with multiple free carboxylic acids in its polymeric structure, find widespread use in drug functionalization [17]. Table 6 presents the most frequently employed carbohydrates for coating Au NPs in biological applications.

### 6.7. Drug Small, Medium and Big Molecule Directed Au NPs

The economic viability of synthesizing small molecules presents significant advantages, demonstrating increased stability compared to previously discussed coating types. Small molecules, owing to their size, facilitate efficient delivery of payloads into tumors. Some molecules, due to their reduced size, necessitate conjugation through linkers such as PEG or polyethyleneimine via amide bonds to attach to Au NPs; examples of functionalization through electrostatic interactions have also been documented [1,17].

The field of nanotechnology has proven invaluable for various applications, including drug delivery, diagnostics and therapeutics, thanks to its special optical and imaging properties, as well as its highly tunable nature [222]. Numerous agents for leveling and stabilizing the surface functionalization of Au NPs have been reported, encompassing thiol/thiolated groups, disulfides, carboxylate groups, amines, hydroxyl compounds, surfactants and phosphate-based chelating agents. Thiol-protected Au NPs, due to their desirable stability and ease of control, characterization and functionalization, exhibit robust Au-S bonds. Concurrently, heteroatoms such as nitrogen (N), oxygen (O) and phosphorus (P) in carboxylate, amine and hydroxyl compounds, and phosphate-based capping agents and stabilizers, engage in electrostatic interactions and surface functionalization with chelator agents, and GDPs [223,224].

Au NPs can be directly linked to small molecules, such as certain drugs or antibiotics, through physical absorption or covalent or ionic bonding. Methotrexate (MTX), an analog of folic acid, stands out as a widely used molecule with demonstrated enhanced cytotoxic activity when conjugated with Au NPs compared to its free state [1].

Attributing antibacterial properties to Au NPs can be achieved through Au-S binding. In 2010, Au-DAPT, functionalized Au NPs with 4,6-diamino-2-pyrimidinethiol (DAPT), demonstrated antibacterial activity against multidrug-resistant Gram-negative bacteria, highlighting the synergistic effect of the components [97]. Au NPs exhibit broad-spectrum antibacterial activities, even against superbugs resistant to most antibiotics, achieved through surface functionalization with an N-heterocyclic molecule [97]. Physical adsorption serves as an additional surface modification method for Au NPs, with pharmaceutical intermediates such as 7-aminocephalosporanic acid (7-ACA), 6-aminopenicillanic acid (6-APA) and 7-aminodeacetoxycephalosporanic acid (7-ADCA) adsorbing onto the surface through physical adsorption between amino and gold groups [97].

Surface functionalization of Au NPs with diverse molecules holds promise for the development of novel antimicrobial materials. Meanwhile, further investigation is essential to understand the effects of different sizes, shapes and surface properties of Au NPs on their antimicrobial properties. Table 7 presents all types of drugs, large and small molecules, used to coat AuNPs for biological applications. 

### 6.8. Polymer and Bio-Functionalized Au NP

The synthesis of water-soluble Au particles stabilized with starch dates back to Helcher’s treatise in 1718, marking the inception of the study of polymer-stabilized Au nanoparticles (P-Au NPs) [283]. P-Au NPs, a subject of ongoing nanotechnological exploration, hold potential for applications in drug delivery. Commonly employed synthetic routes for P-Au NPs include “direct synthesis”, “grafting to” and “grafting from” methods [284].

In the majority of cases, end-functional polymers or selective block copolymers are either physically or chemically adsorbed onto the AuNP surface. This process results in the formation of densely packed surface-tethered polymer chain assemblies, commonly referred to as polymer brushes. Notably, the polymer coating of these AuNPs typically consists of a single type of polymer [285].

P-Au NPs are frequently coated with diverse polymers, such as polyethyleneimine [106,286,287], chitosan [2,187,202], PVA [5] and Pluronic hydrogel [20], among others. In the realm of cancer chemotherapy, polymeric ligands offer several advantages, including prolonged stability of Au NPs, modified solubility, enhanced outer surface hydrophilicity, adjusted shell surface density, reduced immunogenicity and improved biocompatibility [288].

Biocompatible polymers, including poly (ethylene glycol), heparin, hyaluronic acid, chitosan, polystyrenesulfonate, polyethyleneimine and xanthan gum, find application in surface modification of Au NPs for various purposes. These modifications serve to increase the capacity of NPs and payloads, facilitating long systemic circulation followed by cellular uptake. This usage positions Au NPs as a promising system for drug/nucleic acid delivery in cancer therapy [288].

**Figure 12:**
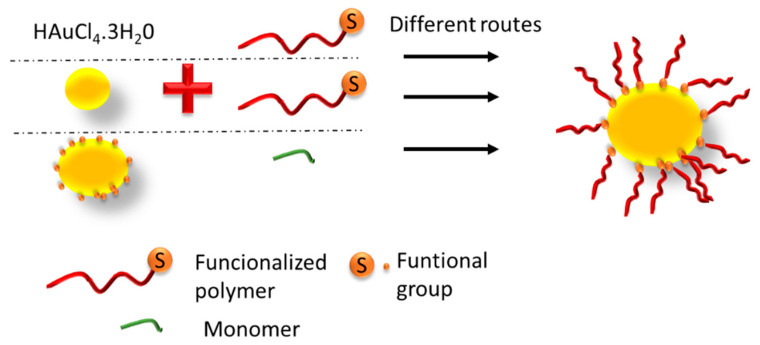
Methods for synthesis of polymeric AuNPs. P-AuNPs have the potential to be used in medicine delivery thanks to advancements in nanotechnology. The “direct synthesis”, “grafting to” and “grafting from” techniques are the main synthetic pathways for P-AuNPs (figure is modified from Muddineti et al.) [288].

The “direct synthesis” method involves the reduction of tetrachloroauric acid (HAuCl4) in the presence of sulfur-terminated polymers, resulting in Au NPs in a single step (Figure 12). On the other hand, “grafting to” refers to the creation of P-AuNPs by attaching functionalized polymers to their surface, utilizing polymers with thiol or amine groups at the beginning, middle or end of the polymer to stabilize Au NPs. The Brust–Schiffrin method outlines this process, wherein the reaction proceeds through ligand substitution [289].

Gold nanoparticles functionalized with biopolymers, particularly those based on polysaccharides, exhibit outstanding biocompatibility and minimal toxicity [96]. The positive charge and hydrophobicity of biopolymers, such as chitosan, contribute to the effective stabilization of Au NPs, mitigating their pronounced agglomeration tendency and fostering electrostatic repulsion [96]. Moreover, polymers not only deter the aggregation of Au NPs but also possess the capability to modulate their morphology by controlling reaction conditions.

Chitosan, renowned for its abundance and cost-effectiveness, emerges as one of the most widely employed biopolymers, serving dual roles as a stabilizer and a reducing agent. Additionally, macrocyclic supramolecules like cyclodextrin, characterized by unique and size-adjustable cavity structures, find application in AuNP stabilization. The synthesis of P-Au NPs involves the “grafting to” method, wherein functionalized polymers are attached to the NP surface, typically employing polymers with thiol or amine groups [96]. Ligand substitution further propels the reaction. Another approach, known as reverse addition fragmentation chain transfer (RAFT) or “grafting from”, entails polymerization at the Au surface, initiated by a species or chain transfer agent, leading to subsequent polymer chain growth from the AuNP surface [96,288].

Beyond chitosan, various polysaccharides and carbohydrates, such as heparin, serve as effective reducing agents, enhancing biocompatibility, stability and anticoagulant activity of synthesized Au NPs [96]. In Table 8, we provide an overview of key biopolymers and biofunctionalizations extensively employed in biological applications of Au NPs.

### 6.9. Cell-Membrane-Coated Au NPs

Cell-membrane-coated gold nanoparticles (Au NPs) offer a versatile approach for treating various pathologies, leveraging inherent properties inherited from the cells of origin. This innovative technology has been applied to a diverse range of cell types, including red blood cells, platelets, tumor cells, immune cells and various bacteria (Table 9), playing a pivotal role in disease surveillance and prevention [303].

The fabrication of nanoparticles with cell membrane coatings involves several methodologies.

**Figure 13:**
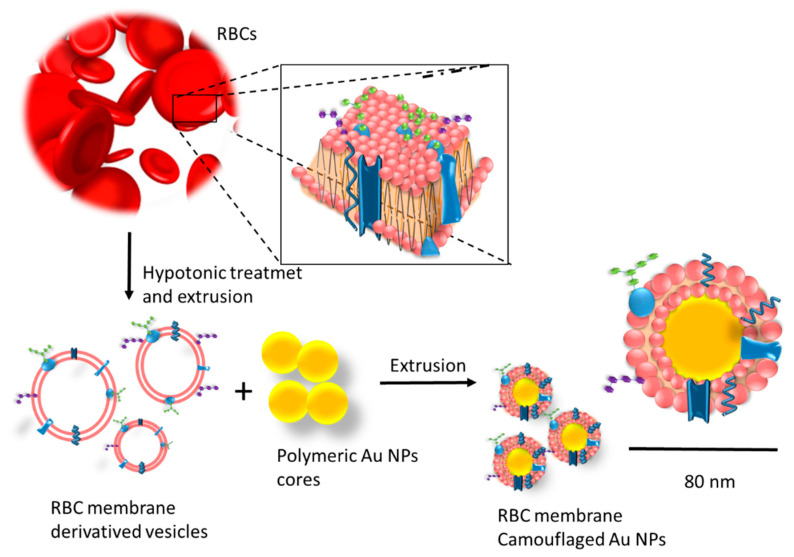
Schematic depicting the fabrication of red blood cell (RBC)-membrane-coated Au NPs (adapted from Hu et al.) [305].

1. Firstly, membrane-coated nanoparticles can be prepared through membrane fusion using physical extrusion, a process akin to liposome synthesis. This technique employs porous membrane materials with uniform pore diameters, facilitating the creation of nanoparticles (Figure 13) [103].
2. Secondly, ultrasonic treatment also induces the spontaneous formation of nanoparticles from core–shell structures enveloped by cell membranes, exhibiting morphologies comparable to those obtained through physical extrusion [304].
3. An alternative and noteworthy approach involves the utilization of microfluidic systems. Reports indicate that combining rapid mixing and electroporation in microfluidic devices can synthesize cell-membrane-coated nanoparticles with enhanced structural integrity and functional reserve, ensuring more efficient production. This cutting-edge technology employs a microfluidic device comprising a Y-type channel, an S-shaped channel mixer and an electroporation area. Through this system, transient pores are generated in cellular membrane memory via electronic pulses, facilitating nanoparticle encapsulation by cell membranes. Notably, this method outperforms alternative strategies by preserving memory and membrane integrity while minimizing protein loss from the membrane surface.

In summary, the use of cell-membrane-coated nanoparticles presents a promising avenue for addressing various pathologies. The diverse synthesis methods, including physical extrusion, ultrasonic treatment and microfluidic systems with electroporation, provide researchers with versatile tools to tailor nanoparticles for specific applications, while also ensuring the preservation of cellular properties critical for therapeutic efficacy [103,303,306].

In view of the multifaceted nature of the challenges faced by cell membrane coating technology, recent studies emphasize the importance of a holistic approach to optimization. This approach involves a comprehensive examination of the manufacturing processes, with a keen focus on identifying and mitigating bottlenecks that impede yield. By strategically addressing these bottlenecks, researchers aim to not only enhance the efficiency of the manufacturing process but also pave the way for a more seamless translation of this technology into diverse biomedical applications [303].

**Table 9:** Cell membrane AuNP coatings. DD—Drug delivery, CT—Cancer treatment, DA—Diagnostic application, BA—Biomedical application.

Coating	Application
EpCam-RPAuNs (Au nanocages (AuNs) encapsulated anti-tumor drug paclitaxel coated by RBC membranes modified by anti-EpCam antibodies)	DD [307]
4t1 (breast tumor cell)	CT [307]
Macrophages	CT [308]
Erythrocytes	DD [309]
Leukocytes	CT [310]
Monocytes	CT [309]
*Escherichia coli*	BA [176]
HUVEC (endothelial cells)	CT [311]
OMVs (bacterial outer membrane vesicles)	BA [311]
RBC (red blood cells)	BA [312]
MPCM-AuNSs (macrophage-cell-membrane-camouflaged AuNS)	BA [313]
THP-1 (human monocyte leukemia cell membrane)	CT [310]
AuNP-pep@Mem	BA [314]
NSC (neural-stem-cell-mediated—intratumoral)	DD [315]
BM-AuNPs (bacterial-membrane-coated gold nanoparticles)	BA [316]
MDA MB-435 (breast tumor cell)	CT [317]
HTC (rat hepatoma)	CT [318]
MCF-7 (breast cancer cell)	CT [318]
RAW264.7 (Macrophage Abelson Murine Leukemia Virus Transformed)	BA [318]
Cell membrane	CT [319]

## 7. Conclusions

The performance of gold nanoparticles (AuNPs) can be significantly adapted and optimized through the appropriate choice of coatings. Each coating introduces unique characteristics that can enhance stability, circulation time, target specificity and other crucial properties of the AuNPs. Selecting the right coating is, therefore, a critical step to achieve the desired functionality and ensure the therapeutic success of the nanoparticles.

- Impact of Coatings on the Optimization of Gold Nanoparticle Performance

Each of the coatings for gold nanoparticles offers unique advantages. However, their application in practical scenarios can also come with challenges or obstacles. Let us delve into the potential obstacles for each coating:

1—PEG: While it offers biocompatibility, it may induce immune reactions after repeated administrations, a phenomenon known as the “accelerated blood clearance effect”.

2—Antibodies: They can be expensive to produce and stabilize. Furthermore, they might not have a long circulation if not properly modified.

3—Proteins and Peptides: Stability can vary and there is potential for degradation, especially in hostile environments like the human body.

4—Amino Acids: Although they are more accessible, they might not offer the same specificity or functionality as other more complex molecules.

5—Microorganisms (DNA, RNA, Aptamers): Stability can be an issue, and there are potential concerns about the immunogenicity of certain sequences.

6—Carbohydrates: Their functionalization might not be as straightforward or effective as other molecules, and there is potential for immune reactions.

7—Drugs (Medications): Controlled drug release can be a challenge, as well as ensuring the drug remains stable and active.

8—Polymers: Biocompatibility can vary, and stabilizing nanoparticles might be challenging depending on the polymer chemistry.

9—Cell Membranes: There is potential for rapid degradation in the body, and concerns about immunogenicity, depending on the origin of the membrane.

In the realm of gold nanoparticle (AuNP) coatings, PEG stands out, particularly in combination with other molecules. PEG, a non-toxic hydrophilic polymer commonly used in pharmaceutical formulations, enhances stability, and reduces nonspecific interactions, prolonging blood circulation. PEGylated AuNPs, such as PEG-SH, offer stability, biocompatibility, prolonged circulation and cytotoxicity. Despite advances in drug delivery systems like liposomes and PEG nanoparticles, their efficiency and yield remain limited.

Recent advancements enable pharmaceuticals to bind to various inorganic nanomaterials, facilitating a reliable drug delivery platform. Gold nanorods (GNRs) exhibit high conjugation efficiency due to their large surface area. While PEG’s effects on stability and circulation are extensively studied, its impact on payload release is not fully understood.

Aptamers, notably associated with chitosan nanoparticles, show promise for targeted drug delivery. However, challenges persist, including quick filtration in the plasma compartment and susceptibility to nucleases affecting aptamer affinity.

DNA duplexes on gold nanoparticles exhibit higher thermal stability than pure duplexes, impacting payload release. The complex interaction between DNA bases and AuNPs, influenced by keto and amino groups, presents challenges in understanding their binding affinity.

RNA therapeutics, including siRNAs, ASOs and mRNAs, have entered clinical trials, benefiting from advanced design and chemistry. Various approaches, such as AVV-based delivery and nanoparticle methods, are explored, with challenges in effective transfection and RNA molecule encapsulation.

Carbohydrates like carrageenan, fucoidan and chitosan, due to their biocompatibility and ease of modification, are frequently used in AuNP coatings for diverse applications. Amino acids, an underutilized option, can reduce toxicity and increase stability.

Protein-AuNP complexes, dynamic and complex entities, depend on factors like surface chemistry and curvature, influencing biodistribution. Peptides, with lower receptor affinity, offer site-specific conjugation advantages, but their short circulatory half-life poses challenges.

Small molecules, though with high tumor penetration, exhibit lower affinity and selectivity. Polymer ligands, with positive charge and hydrophobicity, enhance solution stability and enable additional functionality.

Membrane-coated nanoparticles, using cell membranes like those of red blood cells, offer versatility in immune response modulation and homologous targeting. Challenges include comprehending membrane-coated particle properties and ensuring stability.

AuNPs, while demonstrating potential in pre-clinical studies, face challenges in toxicity assessment, biodistribution and clinical translation. Advances in coatings and controlled release strategies show promise, but careful consideration is necessary for successful therapeutic application.

In conclusion, despite the substantial benefits and cellular versatility of AuNPs in medical applications, challenges persist in toxicity, stability and clinical translation. Ongoing innovations in nanomedicine offer potential, emphasizing the need for meticulous approaches to overcome existing hurdles and enhance therapeutic efficacy. The development of nanocarriers holds promise for future improvements in human health. 

## Figures and Tables

**Table 1:** PEG-derived Au NP coatings. BA—Biomedical application, DD—Drug delivery, BC—Brachytherapy, CT—Cancer treatment, RT—Radiation therapy.

Coating	Application
PEGMA5–DEAMA95–EGDMA15–TG20	BA [67]
O-(3-carboxypropyl)-O″-[2-(3-mercaptopropionylamino) ethyl]-polyethylene glycol (MW 5000, HS-PEG-COOH)	DD [68]
(MW 5000, HS-PEG-NH_2_)	BA [69]
Thiol-terminated polyethylene glycol (5 kDa)	BA [70]
Polyethyleneimine (branched, 25 kDa)	BC [71]
Mercaptopolyethylene glycol monomethyl ether (MeO-PEG-SH) (2 kDa)	BA [72]
Zwitterionic and non-ionic tetraethylene glycol (TEG)	BA [73]
PEG	CT [74]
2-Hydroxypropane-1,2,3-tricarboxylic acid	RT [75]
HS-PEG-Carboxyl	BA [76]
Tirapazamine Modified	BA [77]
Bovine serum albumin (BSA-PEG)	BA [78]
CPP fragments (CALNNPFVYLI, CALRRRRRRRR)	BA [79]
Ranibizumab biosimilar (Mab)/polyethyleneglycol (PEG)-conjugated, PEG chain (5 kDa and 10 kDa)	DD [80]
Dicarboxylic PolyEthylene Glycol (PEG)-600 (PEG)-Bisphosphonate (Bis-PO)	BA [81]
(Methoxy-poly(ethylene glycol)-b-poly((2-dimethylamino) ethyl methacrylate-co-itaconic acid) (mPEG-b-p(DMAEMA-co-IA)	BA [82]
DSPE-PEG2000	DD [83]
Polyethylene glycol (PEG) and functionalized with the boron-rich anion cobalt bis(dicarbollide)	CT [84]
Thiolated polyethylene glycol(PEG-SH, MW5000)	BA [85]
Thiol terminated methoxy polyethylene glycol	DD [86]
Poly(ethylene glycol) thiols (X–PEG–SH, where X = COOH, NH_2_, CH_3_O; MW = 5000 Da)	CT [87]
PEGylated, RGD peptide modified and disulfide cross-linked short polyethylenimines (DSPEIs)	DD [86]
Paclitaxel-PEG 400	DD [68]
Thiolated methoxy polyethylene glycol (HS-mPEG)	BA [1]
PEGylated-poly(DL-lactic-co-glycolic acid)	BA [22]
Dodecyl-PEG	BA [88]
PEG 600	CT [89]
PEG 7.5 k	CT [90]

**Table 2:** Antibody Au NP coatings. CT—Cancer treatment, BA—Biomedical application, DD—Drug delivery.

Coating	Application
Trastuzumab	CT [17]
Cetuximab	CT [17]
EGFR	CT [17]
Anti-transferrin	BA [17]
CD30	CT [17]
MUC-1	CT [17]
hERG1	CT [17]
DR5	CT [17]
TROP-2	CT [17]
CD44	CT [17]
Anti-human IgG	BA [1]
Anti-CD4	DD [19]
Anti-HER2/c erb-2antibody	CT [10]
Anti-EGFR	BA [10]
F19 monoclonal antibodies	CT [10]
Anti-CA15-3-HRP antibody	CT [10]
Her-2/neu antigen	CT [10]
ScFv (single-chain variable fragment)	CT [10]
SM5-1	CT [106]
Rabbit antihuman IgG	CT [107]
Mouse antihuman f-PSA	BA [107]
Anti-HER2	CT [107]
Cetuximab antibody	CT [108]
Anti-interleukin-13 receptor alpha 2 (anti-IL13Ra2)	CT [109]
Listeria monocytogene antibody (mAb-Lis)	BA [110]
Anti-EGFR	CT [111]
4G2	BA [112]
Goat anti-rabbit IgG	BA [113]

**Table 3:** Protein Au NP coatings. DD—Drug delivery, BA—Biomedical application, CT—Cancer treatment, TA—Theranostic application, DA—Diagnostic application.

Coating (Proteins)	Application
Human serum albumin (alb-AuNP)	DD [114]
Luteinizing hormone releasing hormone (LHRH)	BA [47]
Heavy metal binding proteins (HMBPs) in recombinant *Escherichia coli*	DD [115]
Gel from an Aloe vera plant	BA [116]
Inhibitor SMI#9	CT [117]
Polyami-doamine (PAMAM) G4 dendrimer	CT [118]
CBP4	CT [119]
Extract from the alga *Egregia* sp. intrinsically	CT [120]
Hybrid albumin	CT [121]
Bovine serum albumin (BSA)	CT [122]
Bromelain	CT [123]
Chicken egg white (CEW)	BA [124]
FGF1	CT [17]
Lectin Jacalin	CT [17]
T antigen	CT [17]
Apolipoprotein E	DD [114]
Ovalbumin (OVA)	DD [125]
Collagen	TA [22]
Silk fibroin	TA [22]
Wheat-protein-isolate-based polyacrylamide hydrogel	BA [22]
CYT-6091	DA [20]
Tumor necrosis factor-α (TNF-α)	TA [20]
Transferrin	CT [108]
α- and β-integrin	CT [17]
Neuropilin-1	CT [17]
β-galactosidase	DD [1]
Phospho-tyrosine	BA [1]
Glucose-oxidase	BA [126]
Concanavalin A	CT [127]
CD133/AC133	CT [128]
Sericin	BA [129]
Streptavidin	BA [130]
Horseradish peroxidase	CT [131]
Ferritin	BA [132]
Horseradish peroxidase	BA [133]
Tenascin-C	BA [134]
Streptavidin–biotin	BA [135]
Matrix metalloproteinase-9	BA [136]
5′ thiol-ended DNA	CT [137]

**Table 4:** Peptides and Amino Acid Au NP coatings. BA—Biomedical application, DD—Drug delivery, CT—Cancer treatment.

Coating	Application
VG-21	BA [151]
Peptides: CLPSSD, CLPTTD, CLPAAD, CLPVVD, CLPLLD, CLPIID, CLPYYD, CLPWWD, CLPYYD	BA [145]
Hairpin	DD [152]
Aspartate (Asp), glycine (Gly), leucine (Leu), lysine (Lys), serine (Ser), arginine (Arg)	BA [138]
Glucagon-like peptide-1 (GLP-1)	DD [153]
L-aspartate	CT [106]
Lycosin-I	CT [154]
Indolicidin	BA [155]
Plectin-1 (KTLLPTP)	CT [17]
KTLLPTPYC	CT [17]
c(RGD)	CT [17]
CRGDK	CT [17]
GE11 (YHWYGYTPQNVI)	CT [17]
Aspartic acid	DD [1]
Glutamic acid	DD [1]
Phenylalanine	DD [1]
Tryptophan	DD [1]
L-cysteine	DD [1]
Crotamine	DD [19]
Cyclic RGD (cRGD)	DD [47]
Peptide p12 (TSFAEYWNLLSP)	CT [17]
PDC: peptide–drug conjugate containing chlorambucil, melphalan or bendamustine	CT [2]
RGD: arginyl-glycyl-aspartic acid tripeptide	CT [2]
PKC-peptide	CT [10]
Peptide (CCVVGRKKRRQRRRPQGGPLGVEKEKEKEK) and non-responsive peptide (CCVVGRKKRRQRRRPQGGGLPVEKEKEKEK)	CT [156]
RGD-125IPt-PDA-AuNRs	CT [20]
RGD-177Lu-AuNPs	CT [20]
RGD-GdTc-AuNPs	CT [20]
RGD	CT [74]
*Candida antarctica* lipase	BA [157]
Tilapia-scale collagen peptides	BA [158]
RRGTIAFDNWVDTGTRVYDPPPPCC	CT [159]
Primer: 5′- CCGATGCTGTCTTTCGCTGTATCAGGTTCATCACCTGACTCA-3′; reverse primer: 5′-GGCCGCTTTTGCGGGATCTGAGTCAGGTGATGAACCTG ATAC-3′	BA [160]
Lanreotide	CT [161]
NFL-TBS.40–63 peptide (NH2-YSSYSAPVSSSLSVRRSYSSSSGS-CONH2), BIOT-NFL-peptide	BA [162]
TAT.48–60 peptide (BIOT-TAT-peptide; BIOTGRKKRRQRRRPPQ-CONH2)	BA [162]
Vim-TBS.58–81 peptide (BIOT-Vim-peptide; BIOT-GGAYVTRSSAVRLRSSVPGVRLLQCONH2)	BA [162]
N-acetyl-L-cysteine	BA [163]

**Table 5:** Microorganisms, DNA, RNA and aptamers. CT—Cancer treatment, BA—Biomedical application, DD—Drug delivery, DA—Diagnostic application.

Coating	Application
siRNA	CT [186]
anti-STAT3 siRNA	CT [187]
Diosmin with calf thymus DNA (ctDNA)	BA [188]
AS1411 (AS-LAGN)	CT [189]
AS1411—a 26-base guanine (Apt)	DD [17]
sgc8c (Apt)	CT [17]
Nucleolin (apt)	CT [17]
PSMA (apt)	CT [17]
PTK7 (apt)	CT [17]
HER2 aptamer HApt	CT [17]
Antisense oligonucleotides (DNA)	DD [1]
Mimics of tumor suppressive miRNA-miR-205 (DNA)	BA [1]
NU-0129	CT [2]
26-base G-rich DNA oligonucleotide	DA [47]
miR-375	CT [106]
*Mycobacterium*	DD [19]
DNA	BA [190]
ssDNA aptamer	BA [133]
miR-206	CT [191]
Kana aptamer (5′-TGGGGGTTGAGGCTAAGCCG-3′)	BA [192]
Thiol-modified DNA	BA [193]
L-arginine	BA [194]
L-lysine	BA [194]
L-histidine	BA [194]
Anti-HER-2	BA [195]
Tyrosine	BA [196]
5′-Methylene blue (MB)-TCTAACTGCTGCGCCGCCGGGAAAATACTGTACGGTTAGATTTTTTT-(CH2)6-SH-3′.	DA [197]

**Table 6:** Carbohydrate Au NP coatings. DD—Drug delivery, BA—Biomedical application, TA—Theranostic application, DA—Diagnostic application, CT—Cancer treatment.

Coating	Application
Β-cyclodextrin	DD [205]
Lyophilized lipids: 1,2-dipalmitoyl-sn-glycero-3-phosphocholine (DPPC); 2-dipalmitoyl-sn-glycero-3-phospho-(1′-rac-glycerol) (DPPG); 1,2-dipalmitoyl-3-trimethylammonium-propane (DPTAP); 1,2-dipalmitoyl-sn-glycero-3-phosphoethanolamine-N-[methoxy(polyethylene glycol)-5000] (DPPE-PEG5000)	BA [206]
Gellan gum	TA [22]
Extracellular vesicle membranes	D [207]
Lyposomes	CT [208]
Glucose	CT [2]
Lactose	CT [17]
Galectin-1	CT [17]
Guar gum	BA [22]
Gum karaya	BA [22]
Lactobionic acid	DA [106]
1-Thio-D-glucose	CT [209]
Fluoro-6-deoxythio-1-D-glucose	CT [209]
Carrageenan	BA [202]
Alginate	BA [202]
Chitin	BA [202]
Cellulose	BA [210]
Agarose	BA [202]
1,2-Dioleoyl-sn-glycero-3-phosphocholine (DOPC)	BA [211]
1-Dipalmitoyl-sn-glycero-3-phosphocholine (DPPC)	BA [211]
Lipoic acid	BA [212]
Tripeptide glutathione	BA [213]
Thermosensitive lipid	CT [214]
Dextrin	BA [215]
Thioglucose	BA [216]
Saponin niosomes	CT [217]
Carboxymethyl cellulose	CT [218]
Sucrose	BA [219]
G5 PAMAM dendrimers	BA [220]
Gastrodin	BA [220]
Mesquite-gum	BA [221]
Gelatin	TA [22]

**Table 7:** Drug Au NP coatings. BA—Biomedical application, CT—Cancer treatment, DD—Drug delivery, DA—Diagnostic Application, TA—Theranostic application, RT—Radiation therapy.

Coating	Application
Paclitaxel	BA [225]
Catechol	DD [226]
Doxorubicin hydrochloride	CT [123]
Inulin-folate derivative (INU-FA)	DD [227]
1-Dodecanethiol, 1-hexanethio	BA [228]
SN38	CT [229]
GE-genistein,	BA [230]
Cetyltrimethylammonium bromide (CTAB)	DD [231]
11-Mercaptoundecanoic acid (11-MUA)	DD [232]
Gosereline	CT [84]
Imatinib mesylate (IM)	CT [187]
2-Mercapto-1-methylimidazole (MMT)	BA [124]
Phloroglucinol	CT [233]
Lonidamine (LND)	CT [189]
Folic acid	CT [234]
Aminoguanidine	BA [235]
Cationic carbosilane dendrons	DD [236]
Oxaliplatin	CT [107]
Anisamide	CT [17]
α- and β-bicalutamide	CT [17]
Sigma receptor	CT [17]
GPRC6A/MAR	CT [17]
3-Mercaptopropionic acid	DD [1]
4-Mercaptophenol	DD [1]
Docetaxel	CT [17]
Glutathione	CT [2]
AMD3100	DA [47]
Cysteamine–folic acid	DA [237]
XAV939	DD [107]
GSH	CT [108]
Hyaluronic acid (HA)	CT [91]
ssPalmM/DOPE/cholesterol	CT [238]
DMG-mPEG2000	CT [238]
PSS	DD [239]
Lysophosphocholine	DD [240]
Triethylenetetramine	CT [1]
2-Chlorotrityl resin	CT [1]
CALNN	BA [1]
CALNNR8	BA [1]
6-Mercaptopurine-9-b-D-ribofuranoside	CT [10]
N-Succinimidyl 3-(2-puridyldithio) propionate	DD [19]
Methotrexate	DD [241]
Streptomycin	BA [22]
Ampicillin	BA [22]
Kanamycin	BA [22]
Bleomycin	CT [2]
Cationic tetraalkyl ammonium	DD [1]
5-Fluorouracil	CT [27]
Cisplatin	CT [123]
Mitoxantrone	CT [27]
Amoxicillin	BA [22]
Cefaclor	BA [22]
Ciprofloxacin	BA [22]
Gentamycin	BA [22]
Rifampicin	BA [22]
Vancomycin	BA [22]
Methylene blue	BA [22]
Herceptin	CT [10]
Penicillamine	DA [106]
Tiopronin	CT [242]
N-acetyl cysteine	CT [242]
Tamoxifen	CT [107]
Methyl thioglycolate	CT [1]
Biotina	CT [2]
Cetyltrimethyl ammonium bromide	CT [2]
Triphenyl monosulfonate	CT [2]
EGCG	BA [5]
Phthalocyanine	CT [1]
Ethanediamine	BA [47]
Ethanedioic acid	BA [47]
Ce6 carbon dots	TA [47]
2-Mercaptoethylamine	BA [22]
Pyridinium amphiphiles	DD [19]
Amphiphilic triethyleneglycol mercaptan	DD [19]
Fe_3_O_4_	BA [243]
Thiol hydroxyethylmethacrylate (HEMA)	BA [22]
Diatrizoic acid	BA [244]
P-mercaptobenzoic acid	BA [10]
Diethylenetriamine-N,N,N′,N″,N″-pentaacetic acid (DTPA)	BA [106]
[3-Butoxy-2-hydroxypropyl 2-(2,4-dihydroxyphenyl) acetate]	CT [106]
CyNAMLA-381	BA [107]
11-Mercaptoundecanesulfonate	RT [242]
Octanethiol	RT [242]
PH_2_PC_6_H_4_SO_3_H	RT [108]
TPPTS	RT [108]
Platinum	BA [20]
4,6-Diamino-2-pyrimidinethiol (DAPT)	BA [97]
Gemcitabine	CT [17]
CeO_2_	CT [245]
Gadolinium oxide	RT [122]
Tetraethyl orthosilicate (TEOS)	DD [246]
Phosphotungstic acid	BA [247]
3-Aminopropyltriethoxysilane (APTES)	DD [246]
Silica	CT [248]
Cisplatin	BA [249]
4-Selenocyanatoaniline	CT [250]
4,4′-Diselanediyldianiline	CT [250]
N-(4-selenocyanatophenyl)cinnamamide	CT [250]
N-(3-selenocyanatopropyl)cinnamamide	CT [250]
Isoniazid	BA [196]
Quercetin (Qrc, antioxidant)	BA [251]
Sodium citrate	BA [252]
4,6-Diamino- 2-pyrimidinethiol	BA [253]
1,2-Di(9Z-octadecenoyl)-sn-glycero-3-phosphocholine	BA [254]
1,2-Dipalmitoyl-sn-glycero-3-phosphocholine	BA [254]
Tyrosol	BA [255]
Cephradine	BA [256]
cF9-SH	BA [257]
Tannic acid	BA [258]
Protoporphyrin IX	CT [259]
Curcumin	CT [260]
Delafloxacin	BA [261]
Cefotetan	BA [262]
Cefixime	BA [262]
N-succinimidyl 3-(2-pyridyldithio) propionate	BA [263]
Apigenin	BA [264]
Rutin	CT [265]
PAMAM G-4	BA [133]
Dextran/silk sericin	BA [266]
Erythromycin	CT [267]
6-Mercaptopurine	BA [268]
Luteoline	CT [269]
Galangin (3,5,7-trihydroxyflavone)	BA [270]
Kaempferol (3,5,7,4′-tetrahydroxyflavone)	BA [270]
Quercetin (3,5,7,3′,4′-pentahydroxyflavone)	BA [270]
Myricetin (3,5,7,3′,4′,5′-hexahydroxyflavone)	BA [270]
Nitrobenzoic acid	BA [271]
3-Mercaptopropanesulfonate	CT [272]
2-(Diethylamino) ethanethiol hydrochloride	CT [272]
Ciprofloxacin	BA [273]
Levan	CT [274]
(11-Mercaptoundecyl)-N,N,N-trimethylammonium bromide	BA [194]
Lacosamide	BA [275]
L-dopa	BA [276]
Dopamine	BA [276]
β-cyclodextrin	CT [277]
Thioglycolic acid	BA [278]
Hesperetin	CT [279]
Vanillin	BA [280]
Hexadecyltrimethylammonium bromide	CT [137]
5-Aminolevulinic acid	CT [281]
Ziyuglycoside	BA [282]

**Table 8:** Polymer and biofunctionalized Au NP coatings. DD—Drug delivery, BA—Biomedical Application, CT—Cancer treatment, TA—Theranostic application, DA—Diagnostic application.

Coating	Application
Polyethyleneimine, citraconic anhydride modified poly (allylamine) hydrochloride and poly L-arginine	DD [287]
Chitosan	BA [290]
Poly (lactic-co-glycolic acid) (PLGA)	DD [291]
L-α-phosphatidylcholine-hydrogenated soy (HPC), 1.2-dipalmitoyl-d62-sn-glycero-3-phosphocholine (dDPPC)	DD [292]
Polyvinyl-alcohol (PVA)	TA [5]
Polyamidoamine	BA [293]
Amphipathic polymer (hydrophobic PCL/poly(2-hydroxyethyl methacrylate) and hydrophilic poly[2-(2-methoxyethoxy) ethyl methacrylate])	TA [22]
Polypyrrole	TA [10]
Pluronic^®^ F127 (Au-DOX-Gel)	CT [106]
Poly(ethylene glycol)-b-poly(ԑ-caprolactone)	TA [242]
Pluronic hydrogel	CT [20]
PEI AuNPs/51 (polyethyleneimine)	CT [20]
GO-Au-PANI (Polyaniline) core–shell	CT [20]
Au-IPN-pNIPAAm (poly(N-isopropylacrylamide))	CT [20]
PLGA-AuNR-MnO2 (Poly lactic-co-glycolic acid)	CT [20]
Poly (lactic-co-glycolic acid) (PLGA) polymer	DD [291]
PCL	BA [22]
Graphene oxide (GO)-polymer-Au	DA [244]
Hydrophilic polymer, poly(3-dimethylammonium-1-propyne hydrochloride) (PDMPAHCl)	BA [92]
Poly-lysine	BA [1]
Polysarcosine	BA [294]
Liposomes	CT [87]
Poly-(l-lysine)	BA [295]
Poly(diallydimethyl ammonium chloride; PDADMAC)	DD [1]
Poly(sodium-4-stryrenesulfonate; PSS)	DD [1]
Poly(allylamine hydrochloride; PAH)	DD [1]
Hydrogel poly (Nisopropylacryamide) (PNIPAM)	CT [1]
AuroLase	CT [2]
Hydroxylated polyethylene terephthalate (PET)	BA [22]
Polycaprolactone (PCL) gelatin	BA [22]
PVA-BSA	BA [22]
Starch	BA [202]
Chondroitin sulfate	BA [296]
Fucoida	BA [202]
Trimethyl-chitosan	CT [297]
Polyethyleneimine	CT [298]
Pluronic F-127	BA [299]
Polysarcosine–bpolycaprolactone	BA [212]
Polyvinylpyrrolidone	BA [300]
Poly(allylamine–hydrochloride)	BA [210]
Methacrylate monomer	BA [301]
Red blood cell	CT [302]
MCF-7 cancer cell	CT [214]
